# Supplementary material for: Agricultural plant cataloging and establishment of a data framework from UAV-based crop images by computer vision
Source: Gigascience. 2022 Jun 17;11:giac054. doi: 10.1093/gigascience/giac054 (PMC9205758; doi:10.1093/gigascience/giac054)
Supplement: giac054_GIGA-D-21-00328_R3 [file giac054_giga-d-21-00328_r3.pdf]

GigaScience

Agricultural Plant Cataloging and Establishment of a Data Framework from UAV-based Crop Images by Computer Vision

--Manuscript Draft--

**Manuscript Number:**	GIGA-D-21-00328R3
**Full Title:**	Agricultural Plant Cataloging and Establishment of a Data Framework from UAV-based Crop Images by Computer Vision
**Article Type:**	Research
**Funding Information:**	European Agriculture Fund for Rural Development (EP-0004617925-19-001) — Not applicable<br>Bundesministerium für Bildung und Forschung (01DD20001) — Not applicable<br>Deutsche Forschungsgemeinschaft (390732324) — Not applicable

Abstract:

Background

UAV-based image retrieval in modern agriculture enables gathering large amounts of spatially referenced crop image data. In large-scale experiments, however, UAV images suffer from containing a multitudinous amount of crops in a complex canopy architecture. Especially for the observation of temporal effects, this complicates the recognition of individual plants over several images and the extraction of relevant information tremendously.

Results

In this work, we present a hands-on workflow for the automatized temporal and spatial identification and individualization of crop images from UAVs abbreviated as “cataloging” based on comprehensible computer vision methods. We evaluate the workflow on two real-world datasets. One dataset is recorded for observation of Cercospora leaf spot—a fungal disease—in sugar beet over an entire growing cycle. The other one deals with harvest prediction of cauliflower plants. The plant catalog is utilized for the extraction of single plant images seen over multiple time points. This gathers large-scale spatio-temporal image dataset that in turn can be applied to train further machine learning models including various data layers.

Conclusions

The presented approach improves analysis and interpretation of UAV data in agriculture significantly. By validation with some reference data, our method shows an accuracy that is similar to more complex deep learning-based recognition techniques. Our workflow is able to automatize plant cataloging and training image extraction, especially for large datasets.

**Corresponding Author:**	Maurice Günder, M.Sc.<br>University of Bonn: Rheinische Friedrich-Wilhelms-Universitat Bonn<br>Bonn, GERMANY
**Corresponding Author Secondary Information:**	
**Corresponding Author's Institution:**	University of Bonn: Rheinische Friedrich-Wilhelms-Universitat Bonn
**Corresponding Author's Secondary Institution:**	
**First Author:**	Maurice Günder, M.Sc.
**First Author Secondary Information:**	
**Order of Authors:**	Maurice Günder, M.Sc.

Powered by Editorial Manager® and ProduXion Manager® from Aries Systems Corporation

	Facundo Ramón Ispizua Yamati
	Jana Kierdorf
	Ribana Roscher
	Anne-Katrin Mahlein
	Christian Bauckhage
**Order of Authors Secondary Information:**	
**Response to Reviewers:**	Following concerns are no revised:<br><br>* we submitted the dataset to SciCrunch, it is not yes published there, but we have already got an RRID which we also added to the Manuscript under "Availabiliy of Source Code"<br>* the demo dataset is now available on GigaDB, we cited the data with its DOI<br>* we stated the requirements for the conda and docker installation
**Additional Information:**	
**Question**	**Response**
Are you submitting this manuscript to a special series or article collection?	No
**Experimental design and statistics**<br><br>Full details of the experimental design and statistical methods used should be given in the Methods section, as detailed in our Minimum Standards Reporting Checklist. Information essential to interpreting the data presented should be made available in the figure legends.<br><br>Have you included all the information requested in your manuscript?	Yes
**Resources**<br><br>A description of all resources used, including antibodies, cell lines, animals and software tools, with enough information to allow them to be uniquely identified, should be included in the Methods section. Authors are strongly encouraged to cite Research Resource Identifiers (RRIDs) for antibodies, model organisms and tools, where possible.<br><br>Have you included the information requested as detailed in our Minimum Standards Reporting Checklist?	Yes

Powered by Editorial Manager® and ProduXion Manager® from Aries Systems Corporation

**Availability of data and materials**<br><br>All datasets and code on which the conclusions of the paper rely must be either included in your submission or deposited in publicly available repositories (where available and ethically appropriate), referencing such data using a unique identifier in the references and in the “Availability of Data and Materials” section of your manuscript.<br><br>Have you have met the above requirement as detailed in our Minimum Standards Reporting Checklist?	No
If not, please give reasons for any omissions below.<br><br>as follow-up to "**Availability of data and materials**<br><br>All datasets and code on which the conclusions of the paper rely must be either included in your submission or deposited in publicly available repositories (where available and ethically appropriate), referencing such data using a unique identifier in the references and in the “Availability of Data and Materials” section of your manuscript.<br><br>Have you have met the above requirement as detailed in our Minimum Standards Reporting Checklist?<br><br>"	The two datasets reported in the article are not yet published elsewhere, since they are basis of currently ongoing research. This is why we cannot publish the complete dataset. However, to support our results, a subset of the data can be published under an open access license.

Manuscript Click here to access/download;Manuscript;manuscript.pdf

OXFORD (GIGA)n SCIENCE

GigaScience, 2021, 1–21

doi: xx.xxxx/xxxx

Manuscript in Preparation

Research Article

RESEARCH ARTICLE

Agricultural Plant Cataloging and Establishment of a Data Framework from UAV-based Crop Images by Computer Vision

Maurice Günder[1,2,*], Facundo R. Ispizua Yamati[3], Jana Kierdorf[4], Ribana Roscher[4,5], Anne-Katrin Mahlein[3] and Christian Bauckhage[1,2]

[1]Fraunhofer Institute for Intelligent Analysis and Information Systems IAIS, Schloss Birlinghoven, 53757 Sankt Augustin, Germany and [2]Institute for Computer Science III, University of Bonn, Friedrich-Hirzebruch-Allee 5, 53115 Bonn, Germany and [3]Institute for Sugar Beet Research (IfZ), Holtenser Landstraße 77, 37079 Göttingen, Germany and [4]Institute for Geodesy and Geoinformation, University of Bonn, Niebuhrstraße 1a, 53113 Bonn, Germany and [5]Department of Aerospace and Geodesy, Data Science in Earth Observation, Technical University of Munich, Lise-Meitner-Straße 9, 85521 Ottobrunn, Germany

[*]Corresponding author; mguender@uni-bonn.de

Abstract

Background: UAV-based image retrieval in modern agriculture enables gathering large amounts of spatially referenced crop image data. In large-scale experiments, however, UAV images suffer from containing a multitudinous amount of crops in a complex canopy architecture. Especially for the observation of temporal effects, this complicates the recognition of individual plants over several images and the extraction of relevant information tremendously. **Results:** In this work, we present a hands-on workflow for the automatized temporal and spatial identification and individualization of crop images from UAVs abbreviated as “cataloging” based on comprehensible computer vision methods. We evaluate the workflow on two real-world datasets. One dataset is recorded for observation of Cercospora leaf spot—a fungal disease—in sugar beet over an entire growing cycle. The other one deals with harvest prediction of cauliflower plants. The plant catalog is utilized for the extraction of single plant images seen over multiple time points. This gathers large-scale spatio-temporal image dataset that in turn can be applied to train further machine learning models including various data layers. **Conclusions:** The presented approach improves analysis and interpretation of UAV data in agriculture significantly. By validation with some reference data, our method shows an accuracy that is similar to more complex deep learning-based recognition techniques. Our workflow is able to automatize plant cataloging and training image extraction, especially for large datasets.

Key words: UAV imaging; remote sensing; plant identification; plant individualization; precision agriculture

Background

The use of Unmanned Aerial Vehicles (UAVs) is one of the main drivers of modern precision agriculture. Equipped with cameras and other sensors like LiDAR, UAVs can be used for diverse non-invasive in-field analyses and observation tasks [1]. Analyzing and interpreting resulting data with Machine Learn-

Compiled on: May 13, 2022.
Draft manuscript prepared by the author.

Key Points

- Complete automatized workflow from georeferenced images to individual plant images time series
- Parallelized implementation for fast processing
- Enables fast in-field investigation and annotation tasks
- Generation of training data sets for various machine learning tasks
- Basis for a multi-dimensional data framework

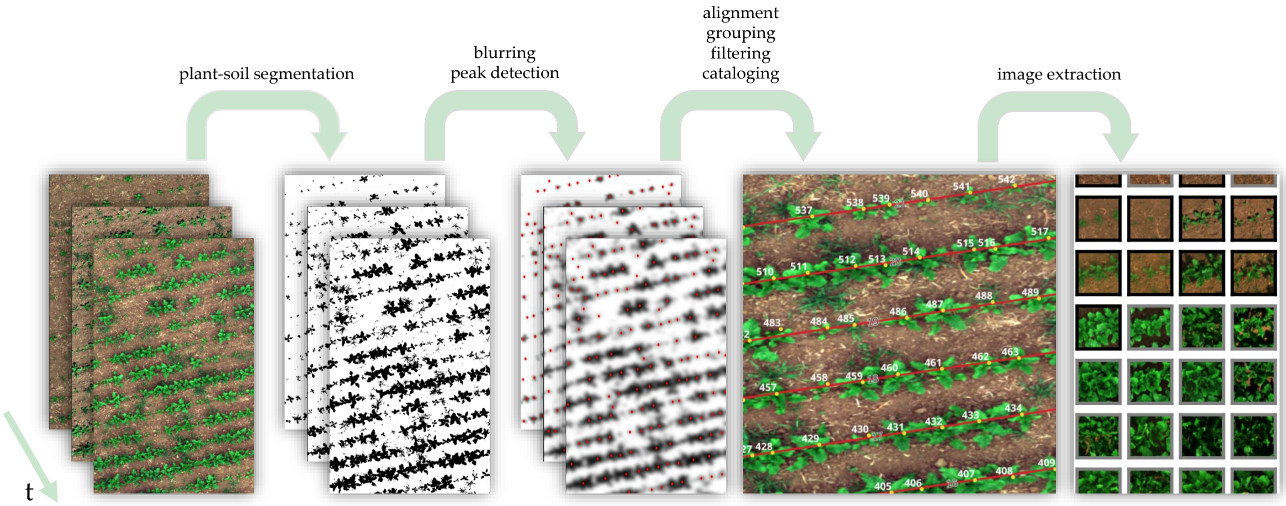

Figure 1. Overview of our proposed plant cataloging and image extraction workflow. The multi-channel UAV images with different acquisition dates are processed for plant-soil segmentation. Afterwards, adaptive Gaussian blur filtering helps to locate the plant positions more precisely via peak finding. Subsequently, the peaks are grouped by time and further manipulation like seeding line detection leads to the plant catalog. Once this catalog is available, one can extract image time series data of single plants.

ing and Pattern Recognition methods has the potential to gain economical and ecological efficiency, which is why computational intelligence is increasingly applied in agricultural contexts [2, 3]. Unlike satellite-based remote sensing [4], UAV-based remote sensing offers low-cost solutions for private usage and individual applications. There are many conceivable use cases like plant species segmentation [5], multi-sensor plant analyses [6, 7, 8], or automated plant counting [9]. Among those, several tasks are based on investigations on individual plants. This is challenging for applications in real-world farming where the fields are densely seeded, in contrast to research cases where plants possibly could have larger distances. We will make use of the cost-effective UAV imaging which enables having multiple images during a complete growing season and combine this information for localizing plants in a spatio-temporal way. Reasons and use cases for cataloging and detecting plants in the field are manifold. On the one hand, it allows determining the number of plants in the field accurately. On the other hand, it enables the extraction of traits of great interest, such as the distance between plants and plant density. Individualization and identification of each plant in the field and the opportunity of retrieving individual plants again in a time series allow generating a vast database of annotations. The availability of this data framework enables to train more robust machine learning models for disease severity analysis, to mention just one possible use case.

To observe individual plants, the exact positions of the plants in the image are required. For the moment, it is possible in two arduous and time-consuming ways: (1) by georeferencing the plants with sub-centimeter accuracy directly on the field by using a GPS receiver with real-time kinematic positioning (RTK) or (2) after image acquisition and orthorectification by manual annotating with a geographic information system (GIS). When taking time series images, the perfect alignment of these images requires the use of well-known and measured points, called ground control points (GCPs) [10]. If the GCPs are correctly georeferenced, they allow allocation of the images not only in a local but also in a global coordinate system, such as WGS84. This enables pixel-precise transferability between images from different locations and time points. The localization of a plant is thus only necessary at one point in time. The determined absolute coordinate remains identical at every point in time, since the plant does not move in space. Thus, a simple extraction of the plant at different points in time is possible.

Overlapping of neighboring plants does not affect localization in this approach if the position is calculated in an early growth stage. If no georeferencing of the data is available, e.g. because no access to measuring devices or GCPs is possible, another possibility is to match the images using plant position detection based on single images. In a further step, those detection points can be registered to each other. However, dealing with overlaps of plants in later developmental stages is challenging in this approach.

Once the different time points are aligned, it is feasible to extract time series for each individual plant. Time series allow the analysis of the plant growth and especially the assessment of the plant traits and their development over time. Based on time series, it is possible to identify the individual development of each plant over time. This spatio-temporal information enables optimization of crop management. Another aspect is the detection of stress factors and disease development, and how it influences the growth of the affected plant over time.

In this work, we introduce a complete workflow for plant identification and individualization—further also referred to as "cataloging"—of single plants. Figure 1 summarizes our proposed workflow with its main steps. We will evaluate the workflow on ground truth data and give possible use case suggestions.

Data Description

In order to show, that our workflow can be used on a variety of UAV image data, we evaluate our approach on two agricultural datasets with different contexts of use. However, the transfer to other datasets and use cases is possible. On the one hand, a dataset from sugar beet for research in the context of Cercospora leaf spot disease spread is used. As this disease is critically affecting the yield of sugar beet farming, several phenotyping studies with UAVs and Unmanned Ground Vehicles (UGVs) have already been done [11, 12]. On the other hand, a dataset is used that monitors the development and harvest time of cauliflower. Such data can be used to extract phenotypic traits such as diameter or height of cauliflower plants or their head [8, 13]. Additionally, plant disease research in general is an excellent use case for this workflow since it requires having individual plant information throughout many growth stages [14, 15, 16]. Thus, a spatio-temporal individualization of the investigated plants is crucial. Especially for large-scale investigations, an approach as automatized as possible is desirable.

Some challenges for both data sets are for example the varying exposure due to the different data acquisition times while the whole growing season and associated different weather conditions. Occurring weeds can have positive as well as negative effects. On the one hand, it simplifies tasks such as registration, since weeds are stationary and thus serve as markers. On the other hand, the growth of weeds affects tasks such as plant detection, since a distinction must be made beforehand between weeds and crops. Another challenge occurs when the plants are so large that the canopy of the plants is closed. This complicates the separation of different plant instances and the search for distinctive points in the field, which is often made possible by the contrast between soil and plants.

Sugar beet dataset

The sugar beet data set was used to develop and evaluate our workflow steps which consists of multispectral drone (UAV) images over time.

Figure 2 shows the location of the plots in the trial field. It was conducted in 2020 near Göttingen, Germany (51°33′N 9°53′E) on a weekly basis at 24 dates during the complete growing season from May 7th to October 12th, 2020. On these, one variety of sugar beet plants that is susceptible against CLS was selected—the *Aluco*[1] variety. The sugar beet was sown on April 6th, 2020, with an inter-row distance of 48 cm, an intra-row distance of 18 cm, and an expected seeding rate of approximately 110 000 plants per hectare.

For the aerial data collection, the quadrocopter *DJI Matrice 210*[2] was used. The position of the UAV during the flight was determined by GPS and GLONASS and corrected by the *D-RTK 2 Mobile Station*[2]. The camera mounted is a *Micasense Altum* multispectral camera[3] with 6 bands: blue (475 nm center, 32 nm bandwidth), green (560 nm center, 27 nm bandwidth), red (668 nm center, 14 nm bandwidth), red edge (717 nm center, 12 nm bandwidth), near infrared (842 nm center, 57 nm bandwidth), and long-wave thermal infrared (8000 nm to 14 000 nm) with a *Downwelling Light Sensor* (DLS2). The ground resolution was set to 4 mm/px. Side-overlap and forward-overlap were set to 75 %. The drone flew in the range around 14 m above ground level with a speed of 0.5 m/s and a resolution of 4 mm × 4 mm per pixel.

1 SESVanderHave
2 SZ DJI Technology Co., Ltd., Shenzhen, China
3 MicaSense, Inc., USA

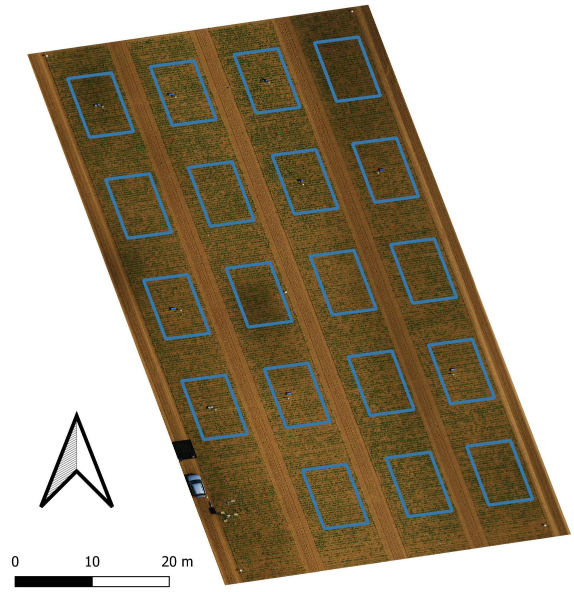

Figure 2. Overview of Cercospora leaf spot experimental field 2020 in Göttingen, Germany. The 19 marked areas correspond to the plots under analysis and undergo different treatments like disease inoculation and fungicide application.The measurements were conducted throughout the full growing season.

In addition, ground control points (GCPs) were set up at the corners and in the middle of the test arrangement for the multitemporal monitoring in the sense of georeferencing and correcting the collected data. *Agisoft Metashape Professional*[4] software has been used to create orthomosaic images. QGIS[5] [17] was used to delimit and select the area to be analyzed, resulting in 19 plots with an equal area of 172 m^2 each.

The experiment present presented three levels: (1) control with fungicide, (2) inoculated with fungicide, and (3) inoculated without fungicide. Plots of inoculated treatments were infected manually with CLS-diseased sugar beet air-dried leaf material.

The first image set was generated in an experimental field focused on detecting of Cercospora leaf spot disease (CLS). CLS is caused by *Cercospora beticola* Sacc. and is one of the most important leaf diseases of sugar beet worldwide. The actual aim of the experiment is to be able to develop models for an accurate and early detection by monitoring of the disease with information from optical and environmental sensors. However, common models are affected by the interaction between plants and the reaction of each plant to the environment. To achieve more accurate and georeferenced information about the plant and its interaction with the pathogen, the need arises to determine the individual position of each plant throughout the development of the growing season.

For the quality evaluation of our workflow, some fields are annotated by human experts, preferably in the earlier stages where the plants are locally distinguishable, by marking the central position of each visible plant.

Cauliflower Dataset

The cauliflower dataset serves for validation of our workflow. We use a subset of the *GrowliFlower* dataset presented in [18]. It consists of a time series of RGB UAV images of a 0.6 ha sized cauliflower field of the Korlanu variety taken on a weekly basis between July, 28th and November 2nd, 2020 near Bonn, Germany (50°46′N 6°57′E). The plants are planted with an intra-row distance of 50 cm and inter-row distance of 60 cm. The cauliflower field was monitored once a week throughout the growing season. As with the sugar beet dataset, the images of the cauliflower dataset were processed into orthophotos using *Agisoft Metashape Professional* software. The flight altitude of the *DJI Matrice 600*[2] hexacopter was 10 m and with a *Sony A7 rIII* RGB camera, we obtain a ground resolution of the orthophotos of roughly 1.5×1.5 mm/px. 21 GCPs were distributed throughout the field and are measured using real-time kinematic positioning (RTK).

The idea behind the acquisition of the dataset is to observe cauliflower plants over its development and to develop models based on the acquired time series that predict, for example, the harvest time of each plant in the field or reflect the maturity state of the plant. Harvesting cauliflower is very laborious, so over several days field workers walk across the field and manually harvest ripe cauliflower heads. In this process, the size of the cauliflower heads is the most crucial plant trait that determines the harvest. Thanks to georeferenced monitoring, it is attempted to help the farmer to optimize his fertilization and pest control as well as to work more economically, in that the field workers already know in advance which plants are to be harvested and do not have to check them individually.

Plant Extraction Workflow

In this section, we will describe the full plant extraction workflow in detail. For each workflow step, we present plots on different excerpts from the sugar beet dataset in order to visualize the results. Figure 1 can be read as a flowchart for the workflow, where the captions on the arrows list the steps top to down.

4 Version 1.6.3.10732
5 Version 3.16

Preprocessing

Some data sets may require preprocessing steps before the workflow can be applied effectively. Firstly, we obviously need georeferenced data in the form of so called orthophotos. These are flattened, distortion-free images of the surface. For our datasets, we generated them from the UAV images by using the software *Agisoft Metashape Professional*, as mentioned above. Secondly, it is beneficial—especially for the seeding line detection that will be discussed later—to crop the orthophotos to the region to be processed by the workflow. Any foreign objects or tramlines should preferably be removed. Foreign objects could have similar color properties than plants and may lead to false recognitions. Furthermore, tramlines may cause irregular seeding line distances, which could lead to inaccurate filtering. Additionally, cropping accelerates the computation since less image data has to be processed by the workflow steps.

Finding Individual Plants

As the first step of plant cataloging, the plant positions are determined for each acquired image, i.e. for each plot at each acquisition date. For this, we perform a segmentation between soil and plants. Common approaches use *Vegetation Indices* (VIs) as a preprocessing for the image information [19]. There are pure RGB-based VIs as well as VIs including multi- or hyperspectral information. Due to the multispectral acquisitions, we have available in the sugar beet dataset, the contrast between plants and soil could be increased compared to pure RGB images. However, our experiments showed that for our dataset, including multispectral information, did not result in better results than with pure RGB information. This is an evidence that our workflow performs well even for use cases where beyond-RGB imaging is not feasible.

Vegetation Index

Using spectral information to condense the information in a one-channel image is the main concept of VIs. Besides reducing the image dimension, the goal behind introducing the VI is to enhance the contrast between soil and plants compared with a standard RGB image, for instance. There are plenty of publications of different vegetation indices so far [20, 21]. However, they all combine different spectral channels by a certain calculation specification. In addition, some indices use (empirically motivated) parameters to further optimize them for different use cases. Since we want to demonstrate that our plant extraction method works for pure RGB information already, we stick to RGB-based VIs. Two indices that turn out to work well in our experiments are the *Green Leaf Index* [22]

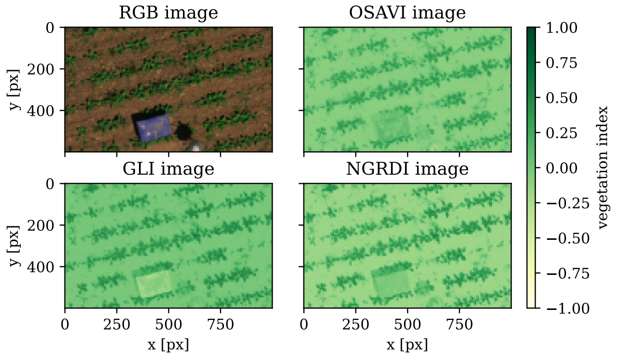

Figure 3. Different VIs for discrimination between plants and soil. The RGB image information (upper left) and three Vegetation Indices are shown. The OSAVI as a multispectral index (upper right) as well as the RGB-only indices GLI (lower left) and NGRDI (lower right) can be calculated for the multispectral sugar beet dataset. Using multispectral information, OSAVI is less susceptible to foreign objects in the field than RGB-only indices.

$$GLI_i = \frac{2G_i - R_i - B_i}{2G_i + R_i + B_i},$$

as well as the *Normalized Green/Red Difference Index* [23]

$$NGRDI_i = \frac{G_i - R_i}{G_i + R_i}.$$

These indices map the information of red (R), green (G), and blue (B) channels of each image pixel i onto a single value in the interval $[-1, 1]$. If there is multispectral data available, nevertheless, one may use the *Optimized Soil Adjusted Vegetation Index* [24] defined as

$$OSAVI_i = \frac{NIR_i - R_i}{NIR_i + R_i + Y},$$

where $Y > 0$ is an empirical parameter. In this work, we use $Y = 0.6$. Although it is not necessary to work with multispectral VIs in this stage, they can further discriminate plants from foreign objects in the UAV image data. Figure 3 shows the mentioned VIs applied on an example image snippet taken from the sugar beet dataset. The remarkable points are that all three VIs can handle shadows fairly well, but the OSAVI is slightly better in ignoring foreign objects.

Note that, surely, also other Vegetation Indices that produce

substantial contrast between plants and soil are conceivable at this point. However, we restrict us to the RGB-only VI images for the further plant finding procedure at this place. They are named as $\mathbf{V}(t)$ for each acquisition date $t \in \mathcal{T}$ in the set of given acquisition dates $\mathcal{T}$. The single pixel values are named by v_i.

Plant-soil Segmentation

In the next step, the soil has to be discriminated from the plant area. Thus, we will perform a segmentation.

As section *Vegetation Index* may already have suggested, the ratio between plant and soil coverage in the images is highly variable for an image series during a complete growing season. Therefore, it is important to keep the growing state—or the plant size—in mind for each image. Thus, a convenient measure is the cover ratio c defined by

$$c = \frac{1}{N_{\mathrm{px}}} \sum_{i}^{N_{\mathrm{px}}} \chi_{\{v_i \geq \vartheta\}}$$

where v_i is the VI value for the i-th of N_{px} total pixels and ϑ is a predefined threshold value. $\chi_{\{\cdot\}}$ is the indicator function, which is 1 if the condition given in the index is met and 0 else. Descriptively, the cover ratio is just the percentage of pixels above threshold.

As threshold technique, we use Otsu's method [25]. The main idea is to find the threshold that minimizes the intra-class variance of two classes to be considered as fore- and background. For intermediately covered fields, this method leads to a reliable discrimination between soil and plants. However, it has weaknesses for the following extreme cases. For very sparsely covered fields ($c < 1\,\%$), Otsu's method usually results in a threshold that is approximately at the most frequent value—i.e. the mode—of the Vegetation Index values. This is because the small amount of "plant pixels" simply disappears in the value distribution, which then has a single peak structure. In those images, the most frequent value is at soil level; thus, we would obtain much noise by using Otsu's threshold and would overestimate the cover ratio calculated by the resulting segmentation mask. We bypass this problem by setting the threshold to the 99-percentile of the VI distribution, which obviously results in a modified cover ratio of exactly 1 %. For cover ratios above circa 75 %, single plants or seeding lines are not properly distinguishable anymore. We therefore do not use those stages for segmentation.

Due to these limitations, we need an initial estimate of the cover ratio in order to classify the images. For the two VIs considered in this work, we investigated good initial thresholds for both datasets. As a result, we set the initial thresholds to $\vartheta_{\mathrm{GLI}} = 0.2$ and $\vartheta_{\mathrm{NGRDI}} = 0$, respectively. For OSAVI, the initial threshold would be set to $\vartheta_{\mathrm{OSAVI}} = 0.25$. With the preliminary estimations for c, we decide which threshold method to use and calculate the final cover ratio for each image. Figure 4 summarizes the findings by some exemplary field sectors during different growing stages.

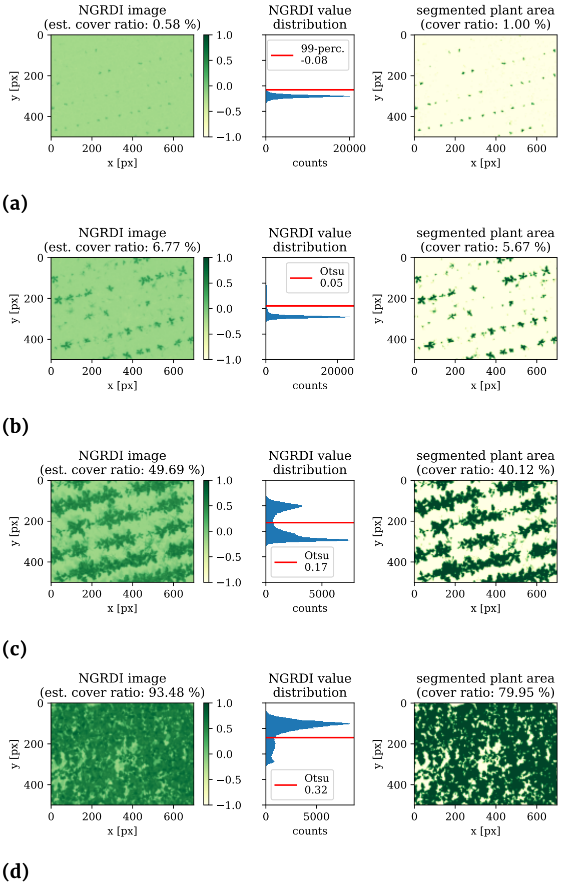

Figure 4. Plant-soil segmentation for different growing stages. The left plots show the NGRDI image. The middle histograms show the distributions of pixel values and the applied threshold for the segmentation—as seen in the right plots. In (a), the estimated cover ratio is below 1 % so that the 99-percentile value is used as threshold. In cases depicted in (b) and (c), Otsu's method is applied. Until roughly 75 % cover ratio, single plants and seeding lines are visible. Images beyond that limit like (d) are not used for further analysis.

Excursus: Growth Function

If enough images during a complete growing season are acquired, we can estimate a functional relation between the single estimated cover ratio. Empirically and plotted against the acquisition date, the cover ratio follows a saturated exponential function until the plants are dying or suffering diseases. From then on, the cover ratio decreases exponentially. This phenomenological considerations motivate the approach for a

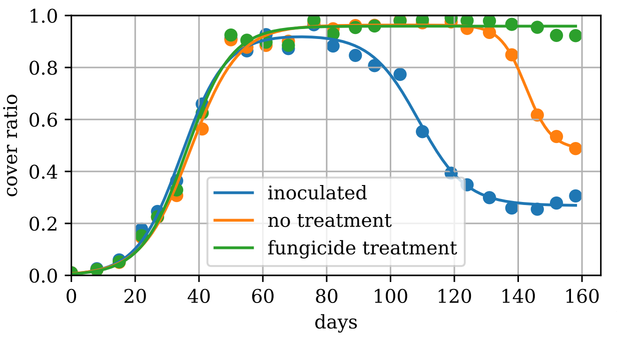

Figure 5. Growth function curve fits. The cover ratio estimates vs. days since first acquisition is fitted with the growth function (1). The shape of the growth function is different for the 3 categories. The blue curve shows an inoculated field. After the growing phase, the dying process due to the disease starts and saturates in the latest acquisitions. In case of the untreated field (yellow curve), the natural dying process is not saturated. In the fungicide treated field shown by the green curve, no dying phase is visible. Thus, the second dying term of the fit function is not present, in contrast to the first two cases.

growth function $f(t)$ defined as:

$$f(t) = \frac{g}{1 + \exp\left(-\lambda_g(t - t_g)\right)} - \chi_{\{d>0\}} \frac{d}{1 + \exp\left(-\lambda_d(t - t_d)\right)} . \quad (1)$$

Basically, we approximate the growing and dying phase as two independent sigmoid functions. The difference of them is the complete growing function. The growing (dying) slope constants λ_g and g (λ_d and d) as well as the corresponding time offsets t_g (t_d) are treated as optimization parameters. By separating cases for positive and non-positive d, one can give the optimization process the option to ignore the dying phase if it is not visible. For the time t, the days since the first acquisition is used. Figure 5 shows the growth function fit for 3 fields with different treatments.

Filtering and Peak Finding

As segmentation masks are calculated, one can find single plants by applying filtering and peak finding techniques. Recap that we have the segmentation masks (cf. *Plant-soil Segmentation*) represented as binary images

$$\mathbf{B}(t) = \left\{\chi_{\{v_i \geq \vartheta(t)\}}\right\}_{i=0}^{N_{\text{px}}}$$

by applying Otsu's or 99-percentile threshold $\vartheta(t)$ for each VI image pixel v_i and acquisition date t. The resulting binary images $\mathbf{B}(t)$ can then be blurred with a Gaussian filter. Mathematically, the images $\mathbf{B}(t)$ are convolved with a 2D Gaussian kernel

$$\mathcal{G}(x, y, t) = \frac{1}{2\pi\sigma^2(t)} \exp\left(-\frac{x^2 + y^2}{2\sigma^2(t)}\right)$$

where $\sigma(t)$ is the bandwidth parameter. x and y represent the 2-dimensional image pixel dimensions. Note the time dependency here, since it makes sense to adapt the bandwidth with the size of the plants—i.e. given by our cover ratio estimate—and by introducing an interval of reasonable bandwidth for the given images $[\sigma_{\text{min}}, \sigma_{\text{max}}]$ in units of image pixels.

As a proxy, the given bandwidth boundaries should roughly represent the size of the plants in the images of the beginning growing stage and maximum growing stage, respectively. Finally, one gets the blurred images $\tilde{\mathbf{B}}(t)$ by convolution ($*$) of the binary images $\mathbf{B}(t)$ with the Gaussian kernel matrix $\mathbf{G}(t)$, hence

$$\tilde{\mathbf{B}}(t) = \mathbf{B}(t) * \mathbf{G}(t) .$$

Subsequently, a simple peak finder is applied to extract the plant centers. The blurring has the effect, that the plants are visible as blurred quasi-circular objects. In this way, the peak finding algorithm detects the plant center rather than individual leaves. To further improve the results of this peak finding, we can set a minimal peak distance and/or intensity to avoid double detection of bigger plants or still visible weed.

Nevertheless, the outcome of this method is highly dependent on choosing the ranges for binary thresholds and Gaussian filter bandwidth correctly. In order to make this more user-friendly and adaptive to new datasets, it is beneficial to use metric units for the parameters and convert them into pixel units by the given geospatial information given. Thus, the workflow user can regard to real-world plant sizes being independent of image resolution. A good choice for σ_{min} and σ_{max} are found to be the radii of a seedling and a fully grown plant, respectively.

Moreover, this method is generally performing significantly better on images with lower plant cover ratios. For higher cover ratios, the plants are no longer spatially divided, which results in misdeterminations or multi-detections. As stated in Figure 4, single plants are hard to discriminate above circa 75 % cover ratio. Thus, we only perform this method on the lower cover ratio images. Figure 6 shows plant detection results for different exemplary growing stages. It happens that also un-

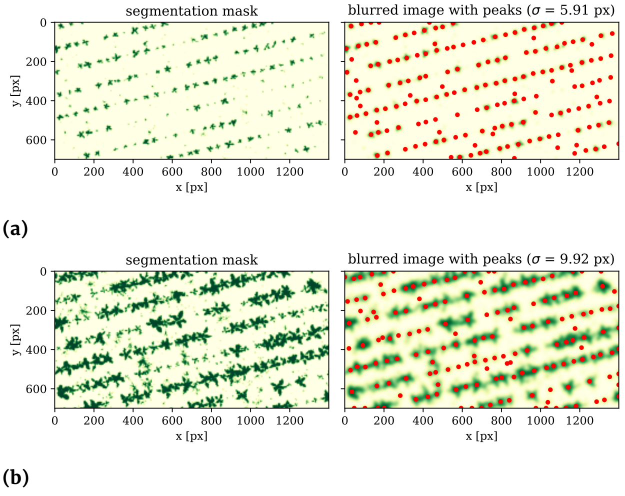

Figure 6. Plant peaks for different growing stages. The left plots show the determined segmentation masks for different growing stages of different fields. On the right, the results of the Gaussian blurring with the adaptive bandwidth and the peak detection (red dots) is shown.

wanted weed is recognized by this method or not every single plant is detected. However, the steps in the following sections minimize those weaknesses by some further filtering and reconstruction methods.

Grouping the Plants

The steps described in chapter *Finding Individual Plants* yield the peak positions for each valid—i.e. with sufficiently low cover ratio—UAV image as pixel coordinates. By using the available geospatial information, the rasterized pixels can be translated into absolute GPS coordinates. Further, we apply a Universal Transverse Mercator (UTM) coordinate system to translate longitude and latitude into a metric scale, e.g. centimeters. This yields metric peak positions

$$\mathcal{P}(t) = \left\{\vec{x}_i(t)\right\}_{i=0}^{N_p(t)},$$

where $\vec{x}_i(t)$ is the position vector of the i-th peak and $N_p(t)$ is the number of detected peaks in the image with acquisition date t. Further, we define $\mathcal{T}$ as the set of all acquisition dates and $\mathcal{T}^* \subseteq \mathcal{T}$ as the set of all acquisition dates where the peak extraction method was performed. The goal of this section is to group this—yet independent—plant positions in a spatial way to identify single plants throughout all images of the whole growing season, even though they might not have been detected in every single image. Therefore, it is mandatory to align the image series in order to correct latent stitching errors or calibration glitches of the UAV's GPS sensor. Additionally, fixed georeference points to align the images to each other—as in the cauliflower dataset can be used. If the plants are seeded in certain line structures—for row crops, cereals is considerably more complex—one can do further steps in finding those lines and filtering "off-line" weed. This procedure is elaborated in the following.

Aligning Plant Positions

In order to avoid confusion of different plant IDs, the metric coordinates $\mathcal{P}(t)$ for each acquisition date t are aligned to each other. Not every single plant is detected in each image. Therefore, the point clouds visualizing the plant positions are not congruent but still highly correlated. Additionally, also weed and other objects that can be observed in the VI images may be included in $\mathcal{P}(t)$. However, we can assume that these incorrectly detected objects are stationary in location as well and, thus, are helpful with the alignment. Afterwards, methods will be described to reduce unwanted objects from the detected peak positions. The main goal of this step is to avoid the largest GPS calibration errors. An application example is shown in Figure 7. In the following, the particular processing steps are described. Furthermore, a pseudocode algorithm for the position alignment can be found in the supplementary material.

Currently, $\mathcal{P}(t)$ contains (absolute) metric coordinates. However, for the next steps, it is convenient to centralize the coordinates by subtracting the centroid of all peak positions. This results in the centralized coordinates

$$\bar{\mathcal{P}}(t) = \left\{\vec{x}_i(t) - \vec{x}_{\text{mean}}\right\}_{i=0}^{N_p(t)},$$
$$\vec{x}_{\text{mean}} := \frac{1}{|\mathcal{T}^*|}\sum_{t\in\mathcal{T}^*}\frac{1}{N_p(t)}\sum_{i=1}^{N_p(t)}\vec{x}_i(t)\,. \qquad (2)$$

Generally, one can apply a transform containing constant shift, shearing, rotation, and scaling by an *affine* transform given by the rule

$$\mathbf{x}'(t) = \begin{pmatrix} T_{00}(t) & T_{01}(t) \\ T_{10}(t) & T_{11}(t) \end{pmatrix}\mathbf{x}(t) + \begin{pmatrix} B_0(t) \\ B_1(t) \end{pmatrix}, \qquad (3)$$

where $\mathbf{x}(t)$ is the $(2 \times N_p(t))$ matrix of the centralized plant positions $\bar{\mathcal{P}}(t)$. We assume that shearing effects are not relevant for the case of GPS calibration issues. By this assumption, one can concretize Equation (3) by only allowing shift, rotation, and

scaling which results in

$$\mathbf{x}'(t) = S(t) \begin{pmatrix} \cos\alpha(t) & -\sin\alpha(t) \\ \sin\alpha(t) & \cos\alpha(t) \end{pmatrix} \mathbf{x}(t) + \begin{pmatrix} B_0(t) \\ B_1(t) \end{pmatrix}. \qquad (4)$$

Thus, we can reduce the generic transformation matrix elements $T_{ij}(t)$ to a scaling factor $S(t)$ and a rotation angle $\alpha(t)$. Initially, the images should be aligned at least roughly so that one can assume that $S(t) \approx 1$ and $\alpha(t) \approx 0°$. The shift parameters $B_0(t)$ and $B_1(t)$ should be in the order of several cm. We will perform what is usually called *registration*. Constraining the transform to the setting in Equation (4), it is also referred to as *rigid registration*. For the optimization, the method of *coherent point drift* (CPD) [26] is applied. Roughly speaking, it is based on a basis point set and a floating one, where the basis point set acts as data points. The floating-point set behaves as the centroids of a Gaussian mixture model (GMM). The objective is to minimize the negative log-likelihood function. At the minimum, the two point sets are considered to be optimally aligned—or registered—to each other. More detailed information is provided in [26].

In order to align all (centralized) *point clouds* $\bar{\mathcal{P}}(t)$ corresponding to the acquisition dates, one has to apply the procedure multiple times for all layers in $\mathcal{T}^*$ step by step. Typically, the lower the cover ratio is, the more peaks are detected. One of the reasons is that the lower blurring bandwidth keep finer structures being visible for the low cover ratios. Therefore, it is useful to sort the acquisitions not by calendar date but by ascending cover ratio as estimated before—either by the growth function fit (cf. *Excursus: Growth Function*), or by the cover ratio estimation (cf. *Plant-soil Segmentation*). For rigid registration, we need a basis point set and a floating point set. As an initial basis set, the point cloud belonging to the image with the lowest cover ratio with acquisition date $t_{\text{init}} \in \mathcal{T}^*$ is chosen, i.e. $\bar{\mathcal{P}}(t_{\text{init}})$. The floating set is the point cloud with the next higher cover ratio. In order to make the alignment robust, only those points of the both sets are considered which have a next neighbor point in the other set within a maximum distance d_{register}.

After performing the registration, the resulting transformation (cf. Equation (4)) is applied on all points of the floating layer. The new basis set is formed by grouping the aligned points that are in the vicinity of each other and calculate their centroid. The resulting set $\bar{\mathcal{P}}_{\text{comb}}$ contains only those group—or cluster—centroids. Points without neighbors inside a given threshold distance d_{group} are considered as new groups.

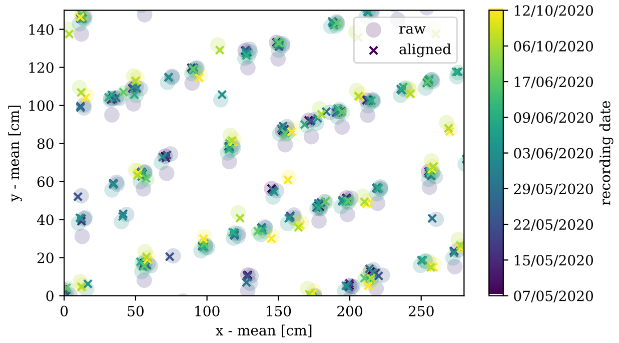

Figure 7. Point cloud alignment. Exemplary snippet from the application on peak positions. The color encodes the chronologically sorted acquisition date. Since the alignment procedure iterates over the layers with ascending cover ratio, this is not necessarily the alignment order. Shaded circles show the raw peak position data, whereas the crosses represent the same data after alignment. The data is already pre-aligned quite well so that only minimal improvements can be observed.

This distance should be chosen smaller than d_{register}. Subsequently, the method is repeated with the basis (centroid) set $\bar{\mathcal{P}}_{\text{comb}}$ and the point cloud with the next higher cover ratio as a floating set.

The developed grouping algorithm is applied in the final grouping as well, so we will make a detailed discussion later in section *Linking Individual Plants During Time*.

Recognizing Seeding Lines

The peak positions are aligned to each other, which means that possible image acquisition errors should be mostly eliminated. Usually—like in the given datasets—the plants are seeded in straight, parallel seeding lines. Recognized peaks off those lines can then be considered as weed, other unwanted objects, or miscellaneous noise. Therefore, it is convenient to extract the seeding line positions in order to filter out those. In order to do this, we align the possible seeding lines with the x- or y-axis, since this is not necessarily the case for the GPS based images which are aligned to a geographical coordinate system. In this work, the seeding lines are aligned to be parallel to the x-axis. In contrast to approaches like in [27] that use the image information directly, we use the point cloud information to infer the seeding line positions and angles. Again, a pseudocode algorithm for seeding line recognition is given in the supplementary material.

Let us consider the acquisition dates $\mathcal{T}^*$ for which the peak point clouds are available. The idea of the seeding lines recognition is to apply a rotation transform on the joint point clouds $\bigcup_{t \in \mathcal{T}^*} \bar{\mathcal{P}}_{\text{aligned}}(t) = \bar{\mathcal{P}}_{\text{aligned}}$. The task is to find the right rotation angle α_s. An established method in the field of computer vision to find regular structures like lines and circles in im-

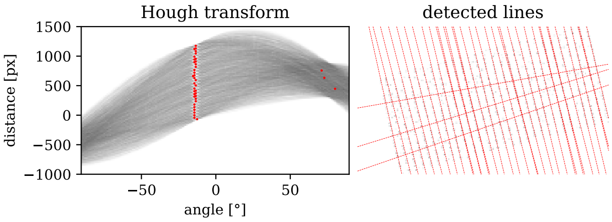

Figure 8. Seeding line rotation angle determination with Hough transform. The left plot shows the Hough transform applied to the rasterized image shown on the right. For reasons of visibility, the right image is rasterized with a 5 times larger bin width than for the actual Hough transformed image. One can see a bunch of nodes in the Hough transform at angles of roughly −14°. Each node corresponds to one found line in the right image. By the vertical distance of the nodes, one can infer the line distances.

ages is the *Hough transform* [28]. It detects points in images that form line structures by transformation of those lines into a feature space (Hough space) consisting of the line angle θ and its minimum distance d from origin. Thus, an infinite line in the (euclidean) image space is mapped to a point in the Hough space. The brute force approach of filling the Hough space is to scan in each non-zero image point a bunch of lines with different angles that intersect in this point. If another point is on this line, one increments the corresponding (θ, d)-bin in Hough space. In the end, the infinite lines that are found in the image are visible by "nodes" in Hough space.

To use this method, we first need to transfer $\bar{\mathcal{P}}_{\text{aligned}}$ into a rasterized image by binning the peaks into a 2D histogram with a fixed bin width. Thus, the bins can be interpreted as image pixels. However, the gained image has a substantially lower resolution than the original image, which makes this method faster than considering the full-resolution plant images as in [27]. Secondly, we initially scan for a fixed amount of angles in $(-90°, 90°]$. This results in a rather rough search of angles, although we expect that all seeding lines are rotated with a common angle. Other randomly found lines in the image can occur, but they are at diffuse angles. The correct seeding line angles is expected to occur regularly so that we can expect a clustering around the correct seeding line angle. Thus, we use the principle of nested intervals: we divide the interval of queried angles into small bins. In the next step, we calculate the histogram of all found angles by using those bins. The diffuse angles will distribute over multiple bins, whereas our seeding line angle should accumulate in one or at least few bins. We take this bin, and maybe the surrounding bins as well, and take these as the new query interval which is then again divided into bins. After several iterations, one finds the common angle of all visible seeding lines α_S. Figure 8 shows the Hough transform applied on a rasterized peak position image.

Since the Hough transform not only yields the line angles but also their distances, one might use this method directly to extract the seeding lines itself. However, it may happen that the Hough transform-based detection misses some less prominent line constellations. This is why we search the seeding line positions again in the point cloud, which is rotated by α_S. Nevertheless, we can use the distance information of the Hough transform results as an estimate for the expected seeding line distances. To have a stable estimate which is robust against missed seeding lines, we use the median seeding line distance for our next step.

We consider only the y-coordinates $\vec{y}_S$ of the rotated point cloud. The principle is to count the points that are inside a certain window with a central y coordinate and a size λ. A scan through all coordinates with a given precision ρ, i.e. the distance between two nearest window centers, results in the sum of included points against the window center position. Finally, one applies a peak finding method to extract the local maxima which represent the (sorted) seeding line positions $\vec{y}^*$. An example plot can be found in the supplementary material. The success of this method is strongly dependent on the right choice of λ and ρ. In order to get this procedure to work stable, it is helpful to set λ and ρ in relation with the seeding line distance estimate of the Hough transform. The peak finding can also be tweaked considering, that one actually knows roughly where the peaks should be located.

Filtering Weed

One benefit of knowing the seeding line positions is that one can effectively filter out recognized objects that are located off the lines which are mainly weed or false detections. Usually, the seeding lines have a regular distance to their neighboring ones so that all distances are approximately by the mean distance. However, if the images contain few irregular seeding line distances, e.g. due to cart tracks in between, it is again helpful to prefer the median distance $\tilde{d}$ over the mean. Additionally, we determine the distance of each point to its next seeding line by

$$d_i = \min |y_i \vec{1} - \vec{y}^*| \,, \tag{5}$$

where y_i is the centralized, aligned, and rotated y-coordinate of the i-th plant position and $\vec{1}^\top = \{1\}^{\dim \vec{y}^*}$. Once all nearest distances d_i are determined, a threshold factor ϑ_d can be

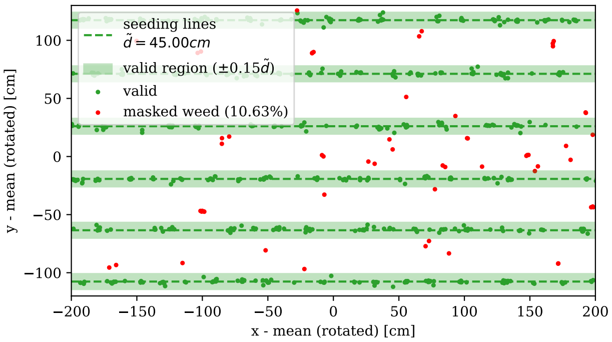

Figure 9. Weed filtering. Excerpt from peak positions where weed filtering is applied. Peaks inside the green shaded valid regions are considered to be valid plants (green dots) whereas peaks being outside are masked as weed (red dots).

set, that specifies, which proportion of the median distance $\tilde{d}$ should be the maximum distance of each plant position to their next seeding line to be considered as valid detection. Hence, the condition is

$$d_i \leq \vartheta_d \tilde{d} \,. \tag{6}$$

The threshold factor is another parameter of the workflow that has to be chosen deliberately. Too small values lead to the incorrect identification of plants as weed, while too large values reduce the filter effect so that much weed is recognized as plants. Figure 9 shows an example weed filtering with threshold factor $\vartheta_d = 0.15$ which turned out to be a good choice for the considered datasets.

Linking Individual Plants During Time

After aligning and filtering the peak positions, we redefine $\mathcal{P}(t)$ to be the aligned, filtered plant positions and $\mathcal{P} := \bigcup_{t \in \mathcal{T}^*} \mathcal{P}(t)$ to be the unified point cloud.

Furthermore, the corresponding image acquisition dates of all plants are summarized in the label vector $\vec{t} \in \mathcal{T}^{*|\mathcal{P}|}$. The goal is to group the point cloud into clusters $\mathcal{G} \subset \mathcal{P}$ and thereby identify single plants recorded in those images where they are detected by the previous methods. An important coordinate of each cluster therefor is its centroid $\vec{\zeta}$ calculated by

$$\vec{\zeta}_i = \frac{1}{|\mathcal{G}_i|} \sum_{\vec{x} \in \mathcal{G}_i} \vec{x} \,, \tag{7}$$

where the $\vec{x}$ are the clusters' point coordinates in $\mathcal{P}$. We call the set of all n_c cluster centroids $\mathcal{C} := \{\vec{\zeta}_i\}_{i=0}^{n_c}$. Furthermore, we can postulate that each cluster contains not more than one member per acquisition date in $\mathcal{T}^*$. Using classical approaches like k-means or even more sophisticated ones like DBSCAN [29] for the clustering, we can hardly make use this knowledge. Those common methods only handle single point clouds without integrating more information on those points. Thus, there may be multiple points from the same acquisition date in one cluster. To integrate the acquisition date information, we use an iterative—or "layer-wise"—approach comparable to a point-to-point registration, similar to the CPD method (cf. *Aligning Plant Positions*). The different acquisition dates are considered as layers of points. Plant detections in plots with low cover ratio are more precise in general, so we start from the layer of the date with lowest cover ratio, as we did for the alignment. Each plant position is considered to be the centroid of a cluster containing—so far—one member. We label those by assigning a unique cluster ID for each centroid. Subsequently, the layer with the second lowest cover ratio is considered. We evaluate a Nearest Neighbor model based on the cluster centroids to get the euclidean distance for each point in the new layer to its next cluster centroid. Next, some decisions have to be made based on this next neighbor distance d. We set a maximum point-centroid distance $d_{\max}$ for points to be considered as members of the corresponding clusters. Thus, if the new candidate is inside this distance ($d \leq d_{\max}$), we label those points with the corresponding cluster ID. It can happen that multiple candidates are inside this distance. We then only register the closest point to the cluster and discard the other candidates. Points outside this distance ($d > d_{\max}$), are assumed to be new clusters labeled new individual cluster IDs. Finally, the cluster centroids are recalculated by including the new candidates to their corresponding clusters. This procedure is repeated iteratively until all layers are processed. In the end, we get clusters with at most $|\mathcal{T}^*|$ members and a label vector $\vec{l}$ containing the corresponding cluster ID for each point. Figure 10 shows an exemplary plot of the cluster centroids that now represent single plants. The IDs are ascending integer values starting from 0. Discarded points get the label −1. The summarizing algorithm, written in pseudocode, can be found in the supplementary material.

Further Filtering and Indirect Detections

With the information of the cluster centroids $\mathcal{C}$ from the results of section *Linking Individual Plants During Time*, we can find a single plant also in images where it is not necessarily detected

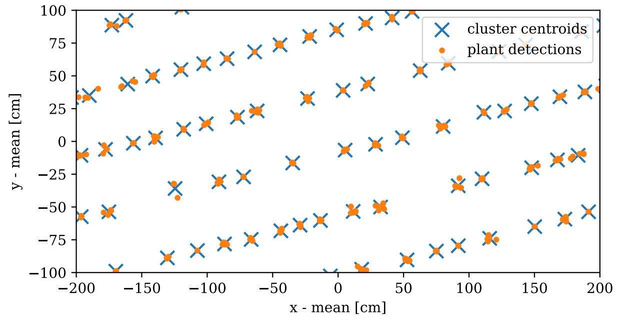

Figure 10. Cluster centroids. Each cluster represents a plant and incorporates the detections in the image series.

since the spatial location is fixed. But first, we start with a somehow "cosmetic" step.

The label numbering in our clustering algorithm is continuous but—due to new clusters starting at different steps—not sorted. However, we may want to have the labels sorted for reasons of clarity and easier retrieval of the plants in in-field operations. A reasonable approach would be to use the seeding line coordinates $\vec{y}^*$ from section *Recognizing Seeding Lines* again in order to determine the seeding line ID for each plant group. The cluster centroids $\mathcal{C}$ are used to calculate the nearest seeding line and assign its ID i_s to each cluster.

$$i_s = \arg\min_{y^* \in \vec{y}^*} |y_c - y^*| \,,$$

where y_c is the y-coordinate of the cluster centroids. Thus, we substitute the initial labels with new sorted labels: primarily sorted by the seeding line ID and secondarily by—in this case for instance—the x-coordinates of cluster centroids. A figure that shows this label sorting for a complete field is shown in the supplementary material.

Moreover, the special point in having the cluster centroid positions is that we can retrieve the plant positions in the high-cover-ratio images—those with acquisition dates $t \in \mathcal{T} \setminus \mathcal{T}^*$—where above methods could not be applied. Additionally, not every plant may be recognized in all analyzed images. In this context, we name the detections by the above methods to be *direct detections*, whereas the reconstructed positions by using the cluster centroids are referred to as *indirect detections*. The aligning algorithm not only gives us the aligned coordinates of the (directly detected) plant positions, but also the transform vectors $\vec{R}(t) := (S(t), \alpha(t), B_0(t), B_1(t))^\top$ for each image[6]. Thanks to the alignments being (rigid) affine transforms, they are invertible. Hence, it is

$$\mathbf{x}(t) = \frac{1}{S(t)} \begin{pmatrix} \cos\alpha(t) & \sin\alpha(t) \\ -\sin\alpha(t) & \cos\alpha(t) \end{pmatrix} \left(\mathbf{x}'(t) - \begin{pmatrix} B_0(t) \\ B_1(t) \end{pmatrix} \right) .$$

by rearranging Equation (4). Thus, we can calculate the plant positions for indirect detections with this inverse transform in order to fill up the point clouds. In order to recover the directly detected plant positions in the individual images, we have to add the calibration error (cf. *Aligning Plant Positions*) again. Please note further that $\mathbf{x}(t)$ are still the centralized UTM coordinates introduced in section *Aligning Plant Positions*. For a complete inverse transform into the GPS coordinate system and the rasterized image pixel system, respectively, we have to add the mean vector $\vec{x}_{\text{mean}}$ again. Finally, the indirect detections at each acquisition date are basically the detected cluster centroids inversely transformed into the coordinate systems of the image data at respective acquisition dates.

With all these above filtering rules, we have a labeled point set where each cluster has exactly $n_t = |\mathcal{T}|$ members, one for each acquisition date $t \in \mathcal{T}$. Finally, we claim the plants to be "cataloged".

Evaluation and Discussion

In this section, we evaluate our results on available ground truth information for both datasets. Furthermore, we introduce two conceivable exemplary application use cases for the workflow.

Validation: Sugar Beet Dataset

With available manually annotated information on the plant positions as a reference, we can validate the above methods. For the sugar beet dataset, there is some ground truth data available which enables us to perform an evaluation of the binary classifiers of *true positive* (*TP*), *true negative* (*TN*), *false positive* (*FP*), and *false negative* (*FN*) detections. Since true negative detections make no sense in this application, measures like the accuracy are not appropriate. Therefore, we focus on two other

6 … as long as the cover ratio is not too high. If it is, however, the alignment can not be performed and the transform vector is the identity transform $\vec{R}(t) := (1, 0, 0, 0)^\top$.

measures called *precision* and *recall* defined by

$$\text{recall} = \frac{TP}{TP + FN}\,, \qquad \text{precision} = \frac{TP}{TP + FP}\,.$$

Descriptively spoken, the recall describes which ratio of really seeded plants our method catches whereas the precision measures the ratio of how many detections of our method indeed are real plants. We want to compare dot-like positions to each other. Thus, we additionally set a tolerance radius of maximum distance between a true plant position and its potential position of detection. We require the plants to be recognized with a maximum tolerance of 8 cm, i.e. the maximum distance between detected plant position and ground truth annotation. The counting of *TP*, *FP*, and *FN* is done thanks to a Nearest Neighbor approach. For each plant detection the ID and the distance to the next true plant position is evaluated. Then we iterate over the true plant positions. For each of them, we look at the distances from the plant detections that were assigned to those corresponding true plants. If there is at least one assignment inside our tolerance, we increment *TP* by one and *FP* by the number of remaining total assignments. If no assignment is inside the tolerance, we increment *FN* by one and *FP* by the number of total assignments. Since every recognized plant position is assigned to exactly one true position, we consider each true and recognized plant by this method. In the end, we obtain the binary classifier counts and can calculate precision and recall. Moreover, our method yield the plant positions by direct and indirect detections. In the ground truth data, the plant is not annotated if it is not visible, for instance in an early image. Since our method uses the inverse transformations of the plant position centroids $\mathcal{C}$, it would already find the plant even if it is not yet visible. Therefore, we ignore all "leading" indirect detections, so all that are earlier than the first direct detection in terms of acquisition date.

The upper plot of Figure 11 shows evaluations of precision vs. recall for field images with available ground truth. Below, for each acquisition date (encoded by the color), an example image is shown. For most of the images, we achieve a precision of at least 90 %. The recall is at least 90 % for most of the images. As we expect, the plant detection gets slightly more inaccurate, the higher the cover ration is due to the lower punctiformity of the plants. However, the early plant detections at lower cover ratios are more confident. This should give the hint that high-cover-ratio images are inappropriate for plant detection and

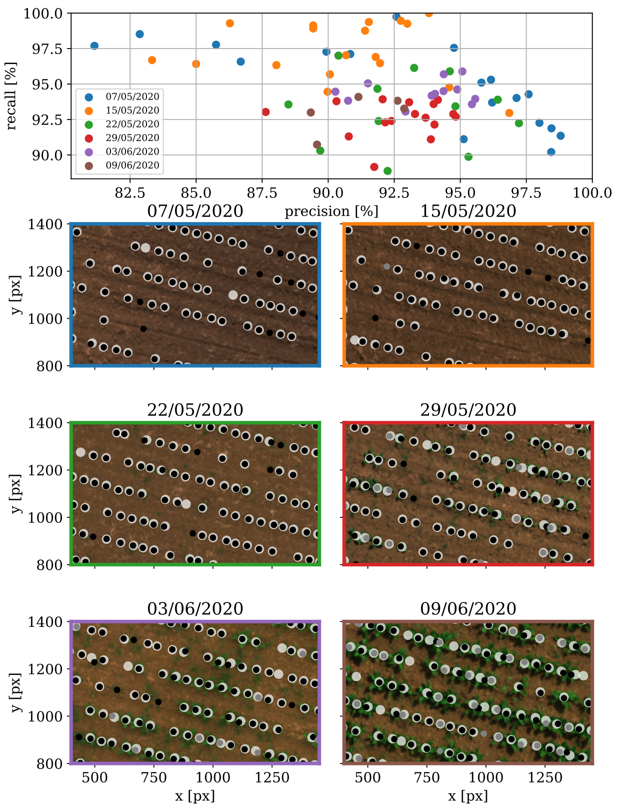

Figure 11. Precision and recall evaluation summary for the sugar beet dataset. The upper plot shows precision vs. recall for all field images with available ground truth data. Colors show the acquisition date of the corresponding image. Below, field excerpts are shown exemplarily—one for each available acquisition date. In these plots, the real plants are marked by white dots. Black dots represent direct detections via our peak detection method. The gray dots are indirect detections by using information of other images of the same field and back transform the positions in each image.

should only be used for the indirect retrieval of already known plant positions at the cluster centroids $\mathcal{C}$.

Generally, the precision is lower than the recall, indicating that our workflow has some false positive plant recognitions. Mostly, those false plants are double recognitions or still remaining weed inside the seeding lines or within the tolerance region defined in the *Filtering Weed* section. Since our method is not sensitive to the actual plant shape like possible deep learning approaches, this remains to be a problem. Our workflow tends to have a higher precision the better the fields are weeded, like in the Cauliflower dataset. The on-line weed may be filtered manually or by further shape-sensitive methods, whereas double detections may be reduced by better clustering parameters.

Validation: Cauliflower Dataset

We evaluate our method on the Cauliflower dataset analogously to the sugar beet dataset described in section *Validation: Sugar Beet Dataset*. The difference is here that there is no "human an-

notated" ground truth data available. However, in the actual use case of the Cauliflower data, a Mask R-CNN [30] trained with single Cauliflower plant images is used to extract the plant position. Our evaluation uses the Mask R-CNN results as reference for our own direct and indirect detections. The Mask R-CNN is applied on a single image (19/08/2020). Nevertheless, we consider the detections to be valid for all acquisition dates to evaluate our date-wise (direct and indirect) detections. As a maximum tolerance distance between our and the reference detections, we choose 12 cm which is roughly the plant radius on the reference detection date. Five dates were available for the evaluation shown in Figure 12. Due to our method, that—as long as being in a reasonable cover ratio range—the plants are detected in each image, our method yields more precise plant positions than the Mask R-CNN approach. This can be crucial for further image extraction of individual plants, where the plants should be centered in each extracted image. Our detections coincide well with the Mark R-CNN detections resulting in a precision above 95 % and a recall above 97 %. In particular, these benchmarks are better than for the sugar beet dataset. This is most likely due to the greater spacing and the resolution of the cauliflower plants compared to the sugar beets.

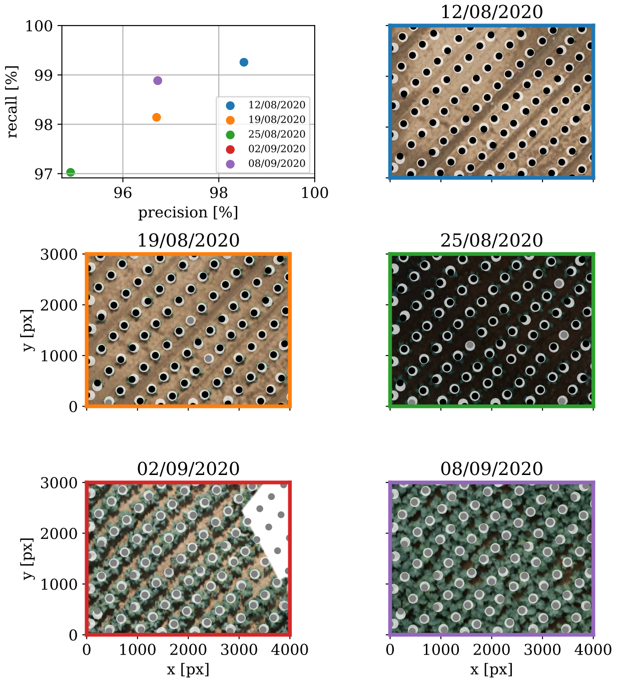

Figure 12. Precision and recall evaluation summary for the Cauliflower dataset. The upper plot shows precision vs. recall for all field images with available reference detection data. Colors show the acquisition date of the corresponding image. Below, field excerpts are shown exemplarily—one for each available acquisition date. In these plots, the real plants are marked by white dots. Black dots represent direct detections via our peak detection method. The gray dots are indirect detections by using information of other images of the same field and back transform the positions in each image. As a reference detection method, plant positions are detected by a Mask R-CNN [30].

Application: In-field Annotations

We have all plant positions available GPS coordinates which enables processing the data with geographic information system applications like *QGIS* [17]. For in-field operations like plant assessment or annotation, the plant catalog can support by providing a structured position sample of interesting plants. Additionally, there are some interesting applications like *QField* [31] that are able to process georeferenced data directly on a smartphone. This is an excellent tool for farmers, who then can do in-field tasks directly "online" using the plant catalog and their current GPS position. Exporting the plant catalog for instance as KML file [32] allows enriching the georeferenced plant catalog with further annotation data like disease severity score, number of leaves, additional images, etc. Figure 13 shows how this could possibly look like for an in-field scenario.

Application: Image Extraction for Disease Severity Classification

An interesting use case of the gathered plant catalog is image extraction. The catalog makes it possible to accumulate a time series of images of individual plants. By inverse transformation of the cluster member coordinates in their respective image pixel-based coordinate system (cf. *Further Filtering and Indirect Detections*) one gets the, say, "original" pixel positions of the plants. Next, we simply define a frame around those pixels and get smaller "tiles". These can then be used for further steps, for instance as a training set for Neural Network architectures as in [33]. The fact that the images are linked to individual plants at multiple dates enables this dataset to be used not only for spatially related but also for time series analyses. Figure 14 shows a few example image tiles for the cauliflower dataset. Other example image tiles for the sugar beet dataset can be found in the supplementary material.

As a brief sample application, we can use the extracted images to automatize the plant health rating by image classification. The rating scale[7] is based on a scale by the corporation *KWS SAAT SE & Co. KGa* (KWS) [34, 35]. Five plant health states are shown and ordered into classes 1, 3, 5, and 9. We complement classes in between, resulting in 10 disease severity degrees from 1 (completely healthy) to 10 (completely dis-

7 german: Bonitur

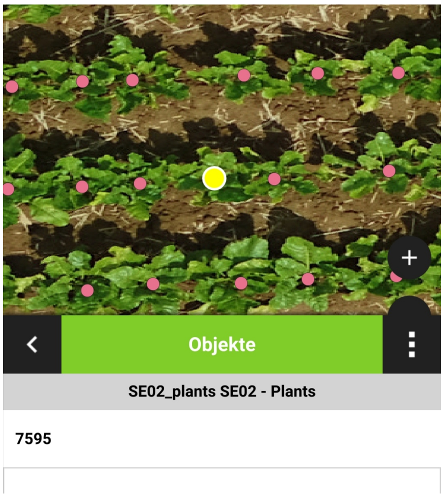

Figure 13. Plant catalog for in-field observations with QField. Exemplary screenshot on an Android smartphone. Detected plants can be accessed individually and various annotations like quality assessments, additional notes, or other images can be added.

eased). A subsample of about 4000 extracted plant images was annotated to train a Convolutional Neural Network (CNN). In order to increase the size of the training sample, we augment the data by sampling further images of the same plants with random shift and rotation. Further augmentation methods like sampling different light conditions are conceivable as well. [36] For the example CNN, we use a standard ResNet-50 model [37] which is pretrained on the ImageNet dataset [38] consisting of RGB images. We adapt the network to our use case (5 channels, 10 output classes) by adding two convolutional layers before the ResNet-50 network successively reducing the 5 channels to 3. After the ResNet-50 layers, we add a dense layer reducing the 2048 classes to our 10 disease severity degrees. The confusion matrix between true and predicted disease severity degrees in Figure 15 shows that the classification is largely possible. However, the discrimination between the first 5 degrees seems to be challenging. Certainly, further research can be done, since this is beyond the scope of this work.

Conclusion

The presented workflow shows a very satisfactory detection performance for both considered datasets. By choosing RGB-only VIs for the peak detection, we demonstrated successfully, that for our plant cataloging, RGB information is sufficient. This could be important for use cases—as shown in the cauliflower dataset—where a multi- or even hyperspectral data acquisition is not feasible for whatever reason. Nevertheless, we may not exclude that the peak detection performance could be improved having available data beyond the optical spectrum.

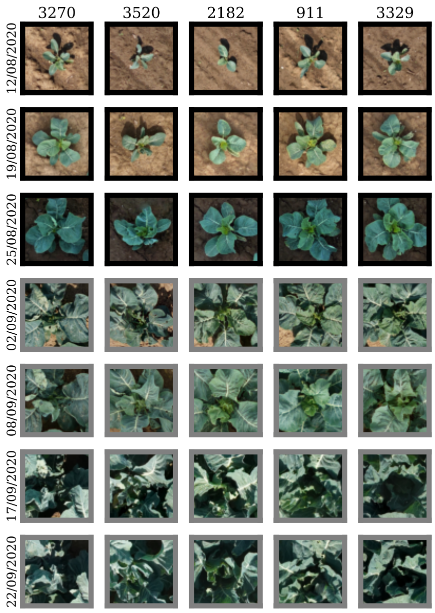

Figure 14. Detected plant positions of cauliflower dataset. 5 randomly picked image series of plant RGB images detected by our method. The acquisition date increases downwards. Black frames annotate direct, gray frames indirect detections.

The high degree of automatization enables the analysis of large-scale data. Particularly for those consisting of multiple smaller fields, like in the sugar beet dataset, many workflow steps can be processed in parallel.

Deep learning based plant recognition approaches have to be trained on the distinct plant sizes if training data is available at all. Unfortunately, for the images with canopy closure ($c > 75\,\%$), both deep learning and our rather "classical" approach will fail, since neighboring plants cannot be distinguished from each other anymore. However, for feasible plant sizes, our approach is mostly agnostic to the plant size and can effectively extract images that can be used to train even better deep learning based plant recognition systems.

By exploiting the geospatial information in UAV images, we can convert between pixel units and metric units like centimeters. From the user's point of view, this makes the workflow very adaptable to other datasets with different plants and seeding conditions. We can directly refer to real-world measures like plant radii or seeding distance without having to consider the image resolution.

High-level deep learning models that are developed recently require large amounts of data, especially for tasks involving image processing like, e.g., classification and instance segmentation with Mask R-CNNs. In principle, non-invasive remote sensing and UAVs enable the application to large-scale agricultural experiments and corresponding data. By automatizing

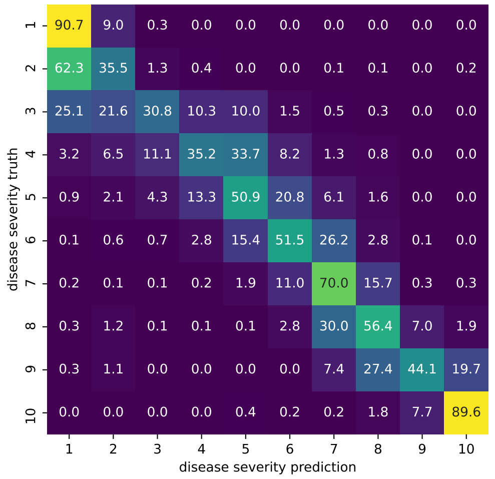

Figure 15. Confusion matrix for the disease severity classification example. The values inside the heatmap are percentages of the corresponding category. Percentages are normalized to the truth, so that all rows sum up to 100 %.

the plant cataloging and providing a data framework, our work helps to exploit the full potential of UAV imaging in agricultural contexts.

Abbreviations

CPD: Coherent Point Drift; CLS: Cercospora Leaf Spot disease; CNN: Convolutional Neural Network; GCP: Ground Control Point; GIS: Geographic Information System (GIS); GLI: Green Leaf Index; GLONASS: Global Navigation Satellite System; GPS: Global Positioning System; LiDAR: Light Detection And Ranging; NGRDI: Normalized Green/Red Difference Index; OSAVI: Optimized Soil Adjusted Vegetation Index; RTK: Real-time Kinematic Positioning; UAV: Unmanned Aerial Vehicle; UGV: Unmanned Ground Vehicle; UTM: Universal Transverse Mercator Coordinate System; VI: Vegetation Index

Competing Interests

The authors declare that they have no competing interests.

Funding

This work has been funded by the Deutsche Forschungsgemeinschaft (DFG, German Research Foundation) under Germany's Excellence Strategy – EXC 2070 – 390732324 and partially by the European Agriculture Fund for Rural Development with contribution from North-Rhine Westphalia (17-02.12.01 - 10/16 – EP-0004617925-19-001). The work is partly funded by the German Federal Ministry of Education and Research (BMBF) in the framework of the international future AI lab "AI4EO – Artificial Intelligence for Earth Observation: Reasoning, Uncertainties, Ethics and Beyond" (Grant number: 01DD20001).

Authors' Contributions

The workflow was designed and implemented by MG. The sugar beet experiments were carried out by FI as well as preprocessing and annotation of the corresponding data. The Cauliflower dataset was provided and preprocessed by JK. MG, FI, and JK drafted the manuscript. CB, AKM, and RR supervised the research. All authors approved the final manuscript.

Availability of Data

A subset of the sugar beet data is available in order to run the workflow and reproduce our results. The data has been uploaded to the GigaScience database (GigaDB), along with snapshots of our code [39]. As mentioned before, the specific use case of the dataset is subsidiary to our workflow. Both datasets are used for ongoing research purposes and therefore cannot be published completely. However, the distributed subset is sufficient to support the results of this article.

Availability of Source Code

The source code of our workflow is available in the following repository:

- Project name: Plant Cataloging Workflow
- GitHub repository: https://github.com/mrcgndr/plant_cataloging_workflow
- RRID: SCR_022276
- Operating system(s): Platform independent (with conda), Linux (with Docker)
- Programming language: Python (3.9 or higher)
- License: Apache License 2.0

References

1. Maes WH, Steppe K. Perspectives for Remote Sensing with Unmanned Aerial Vehicles in Precision Agriculture. Trends in Plant Science 2019;24(2):152–164. https://www.sciencedirect.com/science/article/pii/S1360138518302693.

2. Bauckhage C, Kersting K. Data Mining and Pattern Recognition in Agriculture. KI – Künstliche Intelligenz 2013;27(4):313–324.
3. Kamilaris A, Prenafeta-Boldú F. Deep learning in agriculture: A survey. Comput Electron Agric 2018;147:70–90.
4. Rekha BU, Desai VV, Ajawan PS, Jha SK. Remote Sensing Technology and Applications in Agriculture. In: 2018 International Conference on Computational Techniques, Electronics and Mechanical Systems (CTEMS); 2018. p. 193–197.
5. James K, Bradshaw K. Detecting plant species in the field with deep learning and drone technology. Methods in Ecology and Evolution 2020 Sep;11(11):1509–1519. https://doi.org/10.1111/2041-210x.13473.
6. Huang C, Asner G. Applications of Remote Sensing to Alien Invasive Plant Studies. Sensors 2009 Jun;9(6):4869–4889. https://doi.org/10.3390/s90604869.
7. Savian F, Martini M, Ermacora P, Paulus S, Mahlein AK. Prediction of the Kiwifruit Decline Syndrome in Diseased Orchards by Remote Sensing. Remote Sensing 2020;12(14). https://www.mdpi.com/2072-4292/12/14/2194.
8. Grenzdörffer GJ. Automatic Generation Of Geometric Parameters Of Individual Cauliflower Plants For Rapid Phenoryping Using Dron Images. International Archives Of The Photogrammetry, Remote Sensing & Spatial Information Sciences 2019;.
9. Valente J, Sari B, Kooistra L, Kramer H, Mücher S. Automated crop plant counting from very high-resolution aerial imagery. Precision Agriculture 2020 May;21(6):1366–1384. https://doi.org/10.1007/s11119-020-09725-3.
10. Oniga VE, Breaban AI, Statescu F. Determining the Optimum Number of Ground Control Points for Obtaining High Precision Results Based on UAS Images. Proceedings 2018;2(7). https://www.mdpi.com/2504-3900/2/7/352.
11. Görlich F, Marks E, Mahlein AK, König K, Lottes P, Stachniss C. UAV-Based Classification of Cercospora Leaf Spot Using RGB Images. Drones 2021;5(2). https://www.mdpi.com/2504-446X/5/2/34.
12. Jay S, Comar A, Benicio R, Beauvois J, Dutartre D, Daubige G, et al. Scoring Cercospora Leaf Spot on Sugar Beet: Comparison of UGV and UAV Phenotyping Systems. Plant Phenomics 2020 Aug;2020:1–18. https://doi.org/10.34133/2020/9452123.
13. Drees L, Junker-Frohn LV, Kierdorf J, Roscher R. Temporal Prediction and Evaluation of Brassica Growth in the Field using Conditional Generative Adversarial Networks. arXiv preprint arXiv:210507789 2021;.
14. Mahlein AK, Kuska MT, Behmann J, Polder G, Walter A. Hyperspectral Sensors and Imaging Technologies in Phytopathology: State of the Art. Annual Review of Phytopathology 2018;56(1):535–558. https://doi.org/10.1146/annurev-phyto-080417-050100, pMID: 30149790.
15. Bock CH, Barbedo JGA, Ponte EMD, Bohnenkamp D, Mahlein AK. From visual estimates to fully automated sensor-based measurements of plant disease severity: status and challenges for improving accuracy. Phytopathology Research 2020 Apr;2(1). https://doi.org/10.1186/s42483-020-00049-8.
16. Bock CH, Chiang KS, Ponte EMD. Plant disease severity estimated visually: a century of research, best practices, and opportunities for improving methods and practices to maximize accuracy. Tropical Plant Pathology 2021 Jun;https://doi.org/10.1007/s40858-021-00439-z.
17. QGIS – A Free and Open Source Geographic Information System;. https://qgis.org/.
18. Kierdorf J, Junker-Frohn LV, Delaney M, Olave MD, Burkart A, Jaenicke H, et al., GrowliFlower: An image time series dataset for GROWth analysis of cauLIFLOWER. arXiv; 2022. https://arxiv.org/abs/2204.00294.
19. Hamuda E, Glavin M, Jones E. A survey of image processing techniques for plant extraction and segmentation in the field. Computers and Electronics in Agriculture 2016 Jul;125:184–199. https://doi.org/10.1016/j.compag.2016.04.024.
20. Huete AR, Liu HQ, Batchily K, van Leeuwen W. A comparison of vegetation indices over a global set of TM images for EOS-MODIS. Remote Sensing of Environment 1997;59(3):440 – 451. http://www.sciencedirect.com/science/article/pii/S0034425796001125.
21. Xue J, Su B. Significant Remote Sensing Vegetation Indices: A Review of Developments and Applications. Journal of Sensors 2017;2017:1–17. https://doi.org/10.1155/2017/1353691.
22. Louhaichi M, Borman MM, Johnson DE. Spatially Located Platform and Aerial Photography for Documentation of Grazing Impacts on Wheat. Geocarto International 2001;16(1):65–70. https://doi.org/10.1080/10106040108542184.
23. Tucker CJ. Red and photographic infrared linear combina-

tions for monitoring vegetation. Remote Sensing of Environment 1979;8(2):127–150. https://www.sciencedirect.com/science/article/pii/0034425779900130.

24. Rondeaux G, Steven M, Baret F. Optimization of soil-adjusted vegetation indices. Remote Sensing of Environment 1996;55(2):95–107.
25. Otsu N. A Threshold Selection Method from Gray-Level Histograms. IEEE Transactions on Systems, Man, and Cybernetics 1979;9(1):62–66.
26. Myronenko A, Song X. Point Set Registration: Coherent Point Drift. IEEE Trans Pattern Anal Mach Intell 2010 Dec;32(12):2262–2275. https://doi.org/10.1109/TPAMI.2010.46.
27. Lottes P, Khanna R, Pfeifer J, Siegwart R, Stachniss C. UAV-based crop and weed classification for smart farming. In: 2017 IEEE International Conference on Robotics and Automation (ICRA); 2017. p. 3024–3031.
28. Duda RO, Hart PE. Use of the Hough Transformation to Detect Lines and Curves in Pictures. Commun ACM 1972 Jan;15(1):11–15. https://doi.org/10.1145/361237.361242.
29. Ester M, Kriegel HP, Sander J, Xu X. A Density-Based Algorithm for Discovering Clusters in Large Spatial Databases with Noise. In: Proceedings of the 2nd International Conference on Knowledge Discovery and Data Mining; 1996. p. 226–231.
30. He K, Gkioxari G, Dollár P, Girshick RB. Mask R-CNN. CoRR 2017;abs/1703.06870. http://arxiv.org/abs/1703.06870.
31. QField - A Free and Open Source Geographic Information System;. https://qfield.org.
32. Google Inc , Keyhole Markup Language;. https://developers.google.com/kml/documentation/kmlreference.
33. Yamati FRI, Barreto A, Günder M, Bauckhage C, Mahlein AK. Sensing the occurrence and dynamics of Cercospora leaf spot disease using UAV-supported image data and deep learning. Sugar Industry 2022 Feb;p. 79–86. https://doi.org/10.36961/si28345.
34. Cercospora traits leaflet (*german: Cercospora-Klappkarte*). KWS SAAT SE & Co. KGa;. Accessed: 2021/04/28. https://mediamaster.kws.com/01_Produkte/Zuckerr%C3%BCbe/Infothek-Downloads/cercospora-klappkarte.pdf.
35. Shane WW. Impact of Cercospora Leaf Spot on Root Weight, Sugar Yield, and Purity ofBeta vulgaris. Plant Disease 1992;76(8):812. https://doi.org/10.1094/pd-76-0812.
36. Shorten C, Khoshgoftaar TM. A survey on Image Data Augmentation for Deep Learning. Journal of Big Data 2019 Jul;6(1). https://doi.org/10.1186/s40537-019-0197-0.
37. He K, Zhang X, Ren S, Sun J, Deep Residual Learning for Image Recognition; 2015.
38. Russakovsky O, Deng J, Su H, Krause J, Satheesh S, Ma S, et al., ImageNet Large Scale Visual Recognition Challenge; 2015.
39. Günder M, Yamati FRI, Kierdorf J, Roscher R, Mahlein AK, Bauckhage C, Supporting data for "Agricultural Plant Cataloging and Establishment of a Data Framework from UAV-based Crop Images by Computer Vision"; 2022. http://dx.doi.org/10.5524/102225.

Supplementary Material

Algorithms

Algorithms 1, 2, and 3 show some important steps of our workflow written in pseudocode.

Additional plots

The seeding line recognition based on counting the number of points (plant detections) inside a moving window is shown in Figure 16.

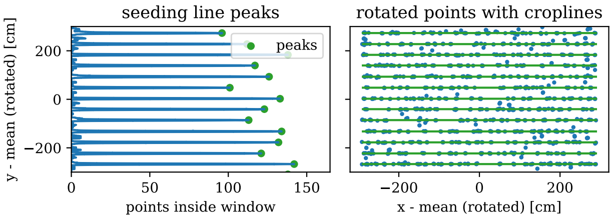

Figure 16. Seeding line recognition. For the seeding line recognition, the points inside a window with the size $\lambda = 32$ px are scanned with a precision of $\rho = 0.5$ px as seen in the left plot. In this example, 29 valid peaks are found by k-means. They represent the center y-coordinates of the seeding lines shown in the right plot with the corresponding (rotated) peak positions.

The label sorting that is done in section *Further Filtering and Indirect Detections* is shown in Figure 17.

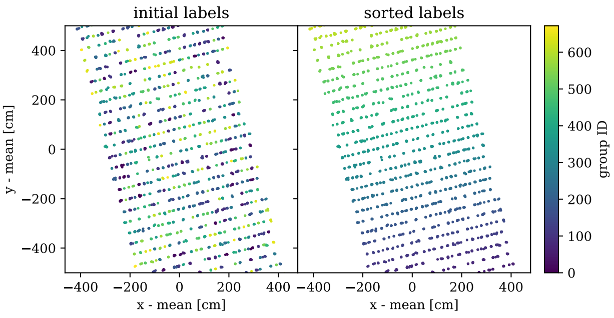

Figure 17. Group label sorting.

Figure 18 shows an image tile plot gathered with our workflow on the sugar beet dataset.

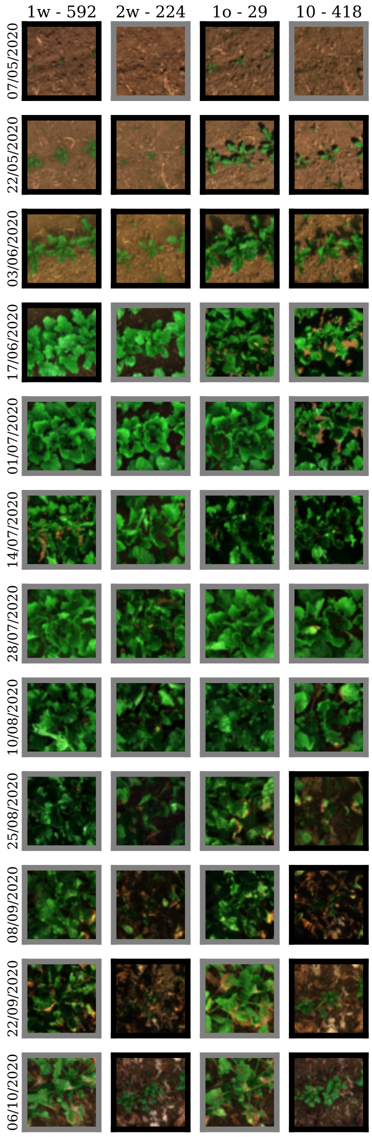

Figure 18. Detected plant positions of sugar beet leaf spot dataset. 4 randomly picked image series of plant RGB images detected by our method. The acquisition date increases downwards. Each second acquisition date is shown. Black frames annotate direct, gray frames indirect detections. The columns are ordered in blocks of 4 examples from inoculated, fungicide-treated, natural and reference fields, respectively.

Algorithm 1 Plant position alignment

Input:
$\bar{\mathcal{P}}(t)$: centralized point clouds ▷ eq. (2)
$\mathcal{T}^*$: acquisition dates sorted by cover ratio in ascending order
$t_{\text{init}} \in \mathcal{T}^*$: acquisition date of initial point cloud with the lowest cover ratio
d_{register}: maximum next neighbor distance between basis and floating layer
$d_{\text{group}} < d_{\text{register}}$: maximum distance from centroid for potential group members
Output:
$\bar{\mathcal{P}}_{\text{aligned}}(t)$: aligned centralized point clouds
$\vec{R}(t) := (S(t), \alpha(t), B_0(t), B_1(t))^\top$: transforms for each layer ▷ eq. (4)
initialize $\vec{R}(t_{\text{init}}) \leftarrow (1, 0, 0, 0)^\top$
initialize $\bar{\mathcal{P}}_{\text{aligned}}(t_{\text{init}}) \leftarrow \bar{\mathcal{P}}(t_{\text{init}})$
initialize $\bar{\mathcal{P}}_{\text{comb}} \leftarrow \bar{\mathcal{P}}(t_{\text{init}})$ ▷ point cloud of cluster centroids (initialized by first layer)
for $t \in \mathcal{T}^* \setminus \{t_{\text{init}}\}$ **do**
    `nn_model_comb` $\leftarrow$ `nearestNeighborModel`$(\bar{\mathcal{P}}_{\text{comb}})$ ▷ fit Nearest Neighbor model to grouped centroid layer
    $\vec{d}_{\text{new}} \leftarrow$ `nn_model_comb.evaluate`$(\bar{\mathcal{P}}(t))$ ▷ get distances for all points of new layer to their next neighboring centroid
    `nn_model_new` $\leftarrow$ `nearestNeighborModel`$(\bar{\mathcal{P}}(t))$ ▷ fit Nearest Neighbor model to new layer
    $\vec{d}_{\text{comb}} \leftarrow$ `nn_model_new.evaluate`$(\bar{\mathcal{P}}_{\text{comb}})$ ▷ get distances for all centroids to their next neighboring point of the new layer
    $\vec{R}(t) \leftarrow$ `rigidCPD`$(\{\bar{\mathcal{P}}_{\text{comb}} \mid d_{\text{comb}} \leq d_{\text{register}}\}, \{\bar{\mathcal{P}}(t) \mid d_{\text{new}} \leq d_{\text{register}}\})$ ▷ perform rigid CPD registration with subsets
    $\bar{\mathcal{P}}_{\text{aligned}}(t) \leftarrow$ `transform`$(\bar{\mathcal{P}}(t), \vec{R}(t))$ ▷ transform complete set using eq. (4)
    $\bar{\mathcal{P}}_{\text{comb}} \leftarrow$ `clusterPoints`$(\bar{\mathcal{P}}_{\text{comb}} \cup \bar{\mathcal{P}}_{\text{aligned}}(t), d_{\text{group}})$ ▷ get cluster centroids (cf. Algorithm 3)
end for

Algorithm 2 Seeding line recognition

Input:
$\mathcal{T}^*$: acquisition dates with given peak positions
$\mathcal{Q} := \bigcup_{t \in \mathcal{T}^*} \bar{\mathcal{P}}_{\text{aligned}}(t)$: centralized aligned point clouds
$\mathbf{q} \in \mathbb{R}^{2 \times n}$, $n = |\mathcal{Q}|$: matrix of point coordinates in point cloud $\mathcal{Q}$ with $\vec{q}_i = (x_i, y_i)^\top$
n_b: number of bins for Hough angles histogram
n_+: number of surrounding bins to respect for nesting iteration
λ: window length for seeding line position scan
ρ: precision for seeding line position scan
Output:
$\vec{y}^*$: seeding line positions
α_s: rotation angle
initialize angle interval $[\alpha_{\text{min}}, \alpha_{\text{max}}] \leftarrow [-90°, 90°]$
$\mathbf{I} \leftarrow$ `2d_histogram`$(\mathbf{q})$ ▷ raster point coordinates into image with given bin width
repeat
    $\mathbf{H} \leftarrow$ `hough_transform`$(\mathbf{I}, [\alpha_{\text{min}}, \alpha_{\text{max}}])$ ▷ perform Hough transform on image in given interval of angles
    $\vec{d}, \vec{\alpha} \leftarrow$ `find_lines`$(\mathbf{H})$ ▷ find nodes in Hough image yielding line distances $\vec{d}$ and corresponding angles $\vec{\alpha}$
    $\mathcal{B} \leftarrow$ set of n_b equal binned intervals in $[\alpha_{\text{min}}, \alpha_{\text{max}}]$
    $\vec{h} \leftarrow$ `histogram`$(\vec{\alpha}, \mathcal{B})$ ▷ $\vec{h}$ counts the number of nodes in the corresponding bin in $\mathcal{B}$
    $\mu = \frac{1}{\dim \vec{\alpha}} \sum_{i=1}^{\dim \vec{\alpha}} \alpha_i$ ▷ take the interval, where most of the nodes are included
    $[\alpha_{\text{min}}, \alpha_{\text{max}}] \leftarrow \bigcup_{i=\max\{1; \arg\max \vec{h} - n_+\}}^{\min\{n_b; \arg\max \vec{h} + n_+\}} \mathcal{B}_i$
until $\mu = \alpha_i \forall \alpha_i \in \vec{\alpha}$
$\alpha_s \leftarrow \alpha_{\text{min}}$
$\mathbf{q_s} \leftarrow \begin{pmatrix} \cos \alpha_s & -\sin \alpha_s \\ \sin \alpha_s & \cos \alpha_s \end{pmatrix} \mathbf{q}$ ▷ rotated point cloud defined analoguously to $\mathbf{Q}$, thus $\vec{q}_{s,i} = (x_{s,i}, y_{s,i})^\top$
initialize point sum vector $\vec{\sigma} \leftarrow \vec{0}$
initialize $i \leftarrow 0$
initialize $y_{\text{test}} \leftarrow \min \vec{y}_s - \lambda$
while $y_{\text{test}} < \max \vec{y}_s + \lambda$ **do**
    $\sigma_i \leftarrow \sum_{l=1}^{\dim \vec{y}_s} I_{\{y_{\text{test}} - \lfloor \frac{\lambda}{2} \rfloor \leq y_{s,l} < y_{\text{test}} + \lfloor \frac{\lambda}{2} \rfloor\}}$
    $y_{\text{test}} \leftarrow y_{\text{test}} + \rho$
    $i \leftarrow i + 1$
end while
$\vec{p}_{\text{peaks}} \leftarrow$ `peakfinder`$(\vec{\sigma})$ ▷ finds local maximum peak positions representing seed line positions
$\vec{y}^* \leftarrow (\min \vec{y}_s - \lambda)\vec{1} + \rho \vec{p}_{\text{peaks}}$ ▷ $\vec{1} := (1, \ldots, 1)^\top$

Algorithm 3 Iterative point clustering

Input:

$\mathcal{P}$: aligned point clouds
$\mathcal{T}^*$: acquisition dates with plant position information sorted by cover ratio in ascending order
$t_{\text{init}} \in \mathcal{T}^*$: acquisition date of initial point cloud with the lowest cover ratio
$d_{\max}$: maximum distance from centroid for potential cluster members

Output:

$\vec{l}$: label vector of cluster ID for each point
$\mathcal{C}$: cluster centroids

initialize $\vec{l} \leftarrow \{-1\}^{|\mathcal{P}|}$ ▷ -1 = not assigned to any cluster
$\{\vec{l} \mid t = t_{\text{init}}\} \leftarrow \{0, 1, \ldots\}$ ▷ assign individual labels for each point of first layer
initialize $\mathcal{C} \leftarrow \{\mathcal{P} \mid t = t_{\text{init}}\}$
for $t' \in \mathcal{T}^* \setminus \{t_{\text{init}}\}$ **do**
    `nn_model` $\leftarrow$ `nearestNeighborModel`$(\mathcal{C})$ ▷ fit Nearest Neighbor model
    $\vec{d}, \vec{\tilde{l}} \leftarrow$ `nn_model.evaluate`$(\{\mathcal{P} \mid t = t'\})$ ▷ get next neighbor distance and ID for all points of respective layer; next neighbor ID corresponds to cluster ID
    initialize $\vec{m} \leftarrow \{1\}^{\dim \vec{d}}$ ▷ vector for new cluster member candidates
    1 = valid member candidate for existing cluster
    0 = valid member candidate for new cluster
    -1 = excluded due to multiple candidates for a single cluster
    $\{m \in \vec{m} \mid d > d_{\max}\} \leftarrow 0$ ▷ assign points with no existing cluster in their vicinity to a new cluster ($m \leftarrow 0$)
    for $\tilde{l}' \in \{\vec{\tilde{l}}\}$ **do** ▷ iterate over the set of cluster IDs in $\vec{\tilde{l}}$
        $\left\{m \in \vec{m} \,\middle|\, d \neq \min\{d \in \vec{d} \mid \tilde{l} = \tilde{l}'\}\right\} \leftarrow -1$ ▷ for multi-assignments: get candidates for one cluster and keep the one with the minimal cluster distance; exclude the others ($m \leftarrow -1$)
    end for
    $\{l \in \vec{l} \mid t = t' \wedge m = 1\} \leftarrow \{\tilde{l} \in \vec{\tilde{l}} \mid m = 1\}$ ▷ assign valid member candidates for existing clusters to respective ones
    $\{l \in \vec{l} \mid t = t' \wedge m = 0\} \leftarrow \{\max(\vec{l}) + 1, \max(\vec{l}) + 2, \ldots\}$ ▷ give new cluster labels to valid member candidates for new clusters
    $\mathcal{C} \leftarrow \emptyset$ ▷ reset clusters before recalculation
    for $l' \in \{\vec{l}\} \setminus \{-1\}$ **do** ▷ iterate over the set of valid cluster IDs in $\vec{l}$
        $\mathcal{G} \leftarrow \{\mathcal{P} \mid l = l'\}$ ▷ consider single cluster with cluster ID l'
        $\mathcal{C} \leftarrow \mathcal{C} \cup \left\{\frac{1}{|\mathcal{G}|} \sum_{\vec{x} \in \mathcal{G}} \vec{x}\right\}$ ▷ recalculate cluster centroids with new cluster assignments (cf. eq. (7))
    end for
end for
