# Supplementary material for: Agricultural plant cataloging and establishment of a data framework from UAV-based crop images by computer vision
Source: Gigascience. 2022 Jun 17;11:giac054. doi: 10.1093/gigascience/giac054 (PMC9205758; doi:10.1093/gigascience/giac054)
Supplement: giac054_Supplemental_File [file giac054_supplemental_file.pdf]

Supplementary Material

Algorithms

Algorithms 1, 2, and 3 show some important steps of our workflow written in pseudocode.

Additional plots

The seeding line recognition based on counting the number of points (plant detections) inside a moving window is shown in Figure 16.

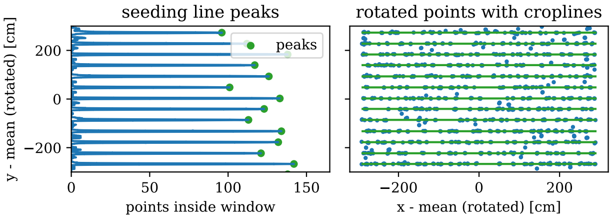

Figure 16. Seeding line recognition. For the seeding line recognition, the points inside a window with the size $\lambda = 32$ px are scanned with a precision of $\rho = 0.5$ px as seen in the left plot. In this example, 29 valid peaks are found by k-means. They represent the center y-coordinates of the seeding lines shown in the right plot with the corresponding (rotated) peak positions.

The label sorting that is done in section *Further Filtering and Indirect Detections* is shown in Figure 17.

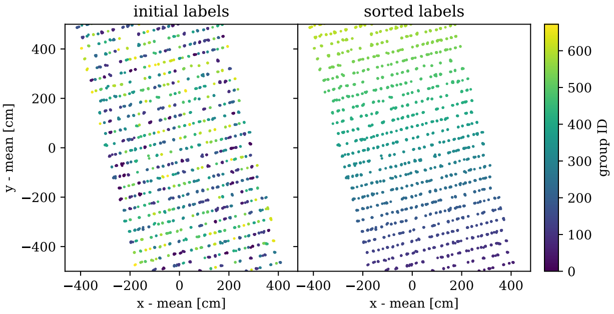

Figure 17. Group label sorting.

Figure 18 shows an image tile plot gathered with our workflow on the sugar beet dataset.

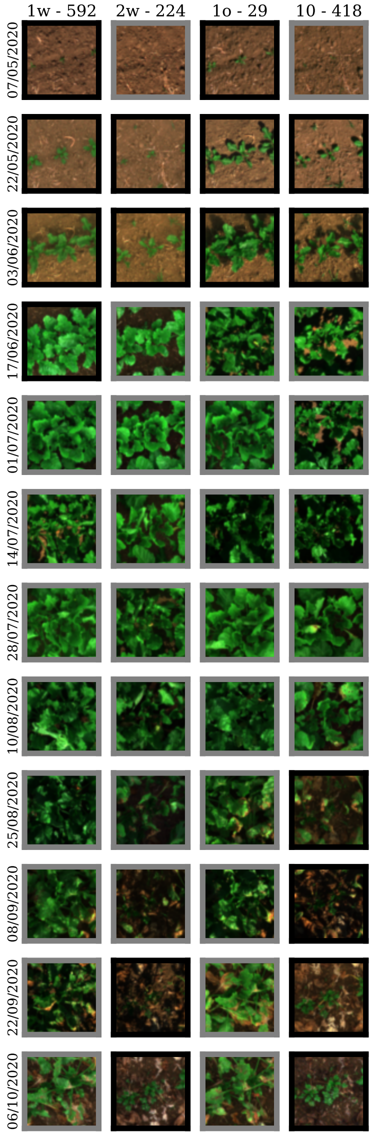

Figure 18. Detected plant positions of sugar beet leaf spot dataset. 4 randomly picked image series of plant RGB images detected by our method. The acquisition date increases downwards. Each second acquisition date is shown. Black frames annotate direct, gray frames indirect detections. The columns are ordered in blocks of 4 examples from inoculated, fungicide-treated, natural and reference fields, respectively.

Algorithm 1 Plant position alignment

Input:
$\bar{\mathcal{P}}(t)$: centralized point clouds ▷ eq. (2)
$\mathcal{T}^*$: acquisition dates sorted by cover ratio in ascending order
$t_{\text{init}} \in \mathcal{T}^*$: acquisition date of initial point cloud with the lowest cover ratio
d_{register}: maximum next neighbor distance between basis and floating layer
$d_{\text{group}} < d_{\text{register}}$: maximum distance from centroid for potential group members
Output:
$\bar{\mathcal{P}}_{\text{aligned}}(t)$: aligned centralized point clouds
$\vec{R}(t) := (S(t), \alpha(t), B_0(t), B_1(t))^\top$: transforms for each layer ▷ eq. (4)
initialize $\vec{R}(t_{\text{init}}) \leftarrow (1, 0, 0, 0)^\top$
initialize $\bar{\mathcal{P}}_{\text{aligned}}(t_{\text{init}}) \leftarrow \bar{\mathcal{P}}(t_{\text{init}})$
initialize $\bar{\mathcal{P}}_{\text{comb}} \leftarrow \bar{\mathcal{P}}(t_{\text{init}})$ ▷ point cloud of cluster centroids (initialized by first layer)
for $t \in \mathcal{T}^* \setminus \{t_{\text{init}}\}$ **do**
    `nn_model_comb` $\leftarrow$ `nearestNeighborModel`$(\bar{\mathcal{P}}_{\text{comb}})$ ▷ fit Nearest Neighbor model to grouped centroid layer
    $\vec{d}_{\text{new}} \leftarrow$ `nn_model_comb.evaluate`$(\bar{\mathcal{P}}(t))$ ▷ get distances for all points of new layer to their next neighboring centroid
    `nn_model_new` $\leftarrow$ `nearestNeighborModel`$(\bar{\mathcal{P}}(t))$ ▷ fit Nearest Neighbor model to new layer
    $\vec{d}_{\text{comb}} \leftarrow$ `nn_model_new.evaluate`$(\bar{\mathcal{P}}_{\text{comb}})$ ▷ get distances for all centroids to their next neighboring point of the new layer
    $\vec{R}(t) \leftarrow$ `rigidCPD`$(\{\bar{\mathcal{P}}_{\text{comb}} \mid d_{\text{comb}} \le d_{\text{register}}\}, \{\bar{\mathcal{P}}(t) \mid d_{\text{new}} \le d_{\text{register}}\})$ ▷ perform rigid CPD registration with subsets
    $\bar{\mathcal{P}}_{\text{aligned}}(t) \leftarrow$ `transform`$(\bar{\mathcal{P}}(t), \vec{R}(t))$ ▷ transform complete set using eq. (4)
    $\bar{\mathcal{P}}_{\text{comb}} \leftarrow$ `clusterPoints`$(\bar{\mathcal{P}}_{\text{comb}} \cup \bar{\mathcal{P}}_{\text{aligned}}(t), d_{\text{group}})$ ▷ get cluster centroids (cf. Algorithm 3)
end for

Algorithm 2 Seeding line recognition

Input:
$\mathcal{T}^*$: acquisition dates with given peak positions
$\mathcal{Q} := \bigcup_{t \in \mathcal{T}^*} \bar{\mathcal{P}}_{\text{aligned}}(t)$: centralized aligned point clouds
$\mathbf{q} \in \mathbb{R}^{2 \times n}$, $n = |\mathcal{Q}|$: matrix of point coordinates in point cloud $\mathcal{Q}$ with $\vec{q}_i = (x_i, y_i)^\top$
n_b: number of bins for Hough angles histogram
n_+: number of surrounding bins to respect for nesting iteration
λ: window length for seeding line position scan
ρ: precision for seeding line position scan
Output:
$\vec{y}^*$: seeding line positions
α_s: rotation angle
initialize angle interval $[\alpha_{\min}, \alpha_{\max}] \leftarrow [-90°, 90°]$
$\mathbf{I} \leftarrow$ `2d_histogram`$(\mathbf{q})$ ▷ raster point coordinates into image with given bin width
repeat
    $\mathbf{H} \leftarrow$ `hough_transform`$(\mathbf{I}, [\alpha_{\min}, \alpha_{\max}])$ ▷ perform Hough transform on image in given interval of angles
    $\vec{d}, \vec{\alpha} \leftarrow$ `find_lines`$(\mathbf{H})$ ▷ find nodes in Hough image yielding line distances $\vec{d}$ and corresponding angles $\vec{\alpha}$
    $\mathcal{B} \leftarrow$ set of n_b equal binned intervals in $[\alpha_{\min}, \alpha_{\max}]$
    $\vec{h} \leftarrow$ `histogram`$(\vec{\alpha}, \mathcal{B})$ ▷ $\vec{h}$ counts the number of nodes in the corresponding bin in $\mathcal{B}$
    $\mu = \frac{1}{\dim \vec{\alpha}} \sum_{i=1}^{\dim \vec{\alpha}} \alpha_i$ ▷ take the interval, where most of the nodes are included
    $[\alpha_{\min}, \alpha_{\max}] \leftarrow \bigcup_{i=\max\{1; \arg\max \vec{h} - n_+\}}^{\min\{n_b; \arg\max \vec{h} + n_+\}} \mathcal{B}_i$
until $\mu = \alpha_i \forall \alpha_i \in \vec{\alpha}$
$\alpha_s \leftarrow \alpha_{\min}$
$\mathbf{q_s} \leftarrow \begin{pmatrix} \cos \alpha_s & -\sin \alpha_s \\ \sin \alpha_s & \cos \alpha_s \end{pmatrix} \mathbf{q}$ ▷ rotated point cloud defined analoguously to $\mathbf{Q}$, thus $\vec{q}_{s,i} = (x_{s,i}, y_{s,i})^\top$
initialize point sum vector $\vec{\sigma} \leftarrow \vec{0}$
initialize $i \leftarrow 0$
initialize $y_{\text{test}} \leftarrow \min \vec{y}_s - \lambda$
while $y_{\text{test}} < \max \vec{y}_s + \lambda$ **do**
    $\sigma_i \leftarrow \sum_{l=1}^{\dim \vec{y}_s} I_{\{y_{\text{test}} - \lfloor \frac{\lambda}{2} \rfloor \le y_{s,l} < y_{\text{test}} + \lfloor \frac{\lambda}{2} \rfloor\}}$
    $y_{\text{test}} \leftarrow y_{\text{test}} + \rho$
    $i \leftarrow i + 1$
end while
$\vec{p}_{\text{peaks}} \leftarrow$ `peakfinder`$(\vec{\sigma})$ ▷ finds local maximum peak positions representing seed line positions
$\vec{y}^* \leftarrow (\min \vec{y}_s - \lambda)\vec{1} + \rho \vec{p}_{\text{peaks}}$ ▷ $\vec{1} := (1, \ldots, 1)^\top$

Algorithm 3 Iterative point clustering

Input:

$\mathcal{P}$: aligned point clouds
$\mathcal{T}^*$: acquisition dates with plant position information sorted by cover ratio in ascending order
$t_{\text{init}} \in \mathcal{T}^*$: acquisition date of initial point cloud with the lowest cover ratio
d_{max}: maximum distance from centroid for potential cluster members

Output:

$\vec{l}$: label vector of cluster ID for each point
$\mathcal{C}$: cluster centroids

initialize $\vec{l} \leftarrow \{-1\}^{|\mathcal{P}|}$ ▷ -1 = not assigned to any cluster
$\{\vec{l} \mid t = t_{\text{init}}\} \leftarrow \{0, 1, \ldots\}$ ▷ assign individual labels for each point of first layer
initialize $\mathcal{C} \leftarrow \{\mathcal{P} \mid t = t_{\text{init}}\}$
for $t' \in \mathcal{T}^* \setminus \{t_{\text{init}}\}$ **do**
  `nn_model` $\leftarrow$ `nearestNeighborModel`$(\mathcal{C})$ ▷ fit Nearest Neighbor model
  $\vec{d}, \vec{\tilde{l}} \leftarrow$ `nn_model.evaluate`$(\{\mathcal{P} \mid t = t'\})$ ▷ get next neighbor distance and ID for all points of respective layer; next neighbor ID corresponds to cluster ID
  initialize $\vec{m} \leftarrow \{1\}^{\dim \vec{d}}$ ▷ vector for new cluster member candidates
    1 = valid member candidate for existing cluster
    0 = valid member candidate for new cluster
    -1 = excluded due to multiple candidates for a single cluster
  $\{m \in \vec{m} \mid d > d_{\text{max}}\} \leftarrow 0$ ▷ assign points with no existing cluster in their vicinity to a new cluster ($m \leftarrow 0$)
  for $\tilde{l}' \in \{\vec{\tilde{l}}\}$ **do** ▷ iterate over the set of cluster IDs in $\vec{\tilde{l}}$
    $\left\{m \in \vec{m} \,\middle|\, d \neq \min\{d \in \vec{d} \mid \tilde{l} = \tilde{l}'\}\right\} \leftarrow -1$ ▷ for multi-assignments: get candidates for one cluster and keep the one with the minimal cluster distance; exclude the others ($m \leftarrow -1$)
  end for
  $\{l \in \vec{l} \mid t = t' \wedge m = 1\} \leftarrow \{\tilde{l} \in \vec{\tilde{l}} \mid m = 1\}$ ▷ assign valid member candidates for existing clusters to respective ones
  $\{l \in \vec{l} \mid t = t' \wedge m = 0\} \leftarrow \{\max(\vec{l}) + 1, \max(\vec{l}) + 2, \ldots\}$ ▷ give new cluster labels to valid member candidates for new clusters
  $\mathcal{C} \leftarrow \emptyset$ ▷ reset clusters before recalculation
  for $l' \in \{\vec{l}\} \setminus \{-1\}$ **do** ▷ iterate over the set of valid cluster IDs in $\vec{l}$
    $\mathcal{G} \leftarrow \{\mathcal{P} \mid l = l'\}$ ▷ consider single cluster with cluster ID l'
    $\mathcal{C} \leftarrow \mathcal{C} \cup \left\{\frac{1}{|\mathcal{G}|} \sum_{\vec{x} \in \mathcal{G}} \vec{x}\right\}$ ▷ recalculate cluster centroids with new cluster assignments (cf. eq. (7))
  end for
end for
