# Supplementary material for: Agricultural plant cataloging and establishment of a data framework from UAV-based crop images by computer vision
Source: Gigascience. 2022 Jun 17;11:giac054. doi: 10.1093/gigascience/giac054 (PMC9205758; doi:10.1093/gigascience/giac054)
Supplement: giac054_Response_to_Reviewer_Comments_Original_Submission [file giac054_response_to_reviewer_comments_original_submission.pdf]

Author's Response To Reviewer Comments

Close

In the following, our response is marked by ">>>".

Reviewer #1: The authors present an automated pipeline for identification of plants in UAV images. Recognition happens over a sequence of images, corroborating the location of plants growing over time. Overall the approach appears to perform well, and has comparable accuracy to an R-CNN based approach, which would be considered standard.

Overall I have a positive impression of the paper, but there are a few concerns I think should be addressed. The main justification of the approach in this paper is that it is effective, and demonstrably similar in performance to deep learning approaches. This is fine, it is reasonable to publish "traditional" image analysis pipelines if they work well. However there were some missing details on the robustness and specifics of the algorithm that would be very useful for readers. I list my main points below:

- As a bottom up pipeline, there are numerous thresholds and defined values that underpin the algorithm. For example, the threshold value for plant vs soil, the function to calculate the width of the gaussian blur, the valid plant region from Figure 9, and so on. While these appear effective, no detail is given on the robustness of the approach to choices of these values. It is possible (without evidence shown) that the algorithm quickly breaks with even small changes to these parameters, if this is the case then there would be a stronger argument for moving towards a deep learning approach, where robustness could be gained from training data. How did you pick these values, what was your experience of picking these values? If another reader comes along with a new dataset, how easy will it be for them to apply your pipeline?
>>> Thanks, the robustness of the parameters is a good point to mention. Indeed, the impact of the parameters is very different. In case of the fixed VI thresholds, these are only initial settings to roughly classify very sparsely and fully covered field. The fine-tuning of this threshold is done by Otsu's method. The initial threshold values for each tested VI by "visual investigation". Nevertheless, we tried to keep parameters equal for both datasets hoping to achieve a rather universal choice for further custom datasets. We added some passages in the manuscript to emphasize this.
Since we assume that remote sensing datasets are georeferenced, we avoid "pixels" as units for regarding parameters and use centimeters instead. This choice is more natural to the user and we mentioned good choices for these parameters with respect to the investigated plants and field circumstances. Therefore, the parameters can be fine-tuned to new datasets more easily, since metric units are independent of image resolution. For instance, we found that a good choice for the min. and max. gaussian width is the plant radius of an early and a fully grown plant, respectively. For the threshold values, we tried different combinations and present our best-choice results. We emphasized above statements in the manuscript and gave more detailed recommendations on parameter choices.

- Related to the above, you do not mention if there are any changes to the experimental parameters moving from one dataset to the cauliflower dataset. If your approach worked immediately with no changes, that would obviously be a postitive!
>>> Yes, in fact, we only need to adjust the "metric" parameters, if switching from sugar beet to cauliflower. That is firstly due to the different plant sizes of cauliflower plants and, secondly, due to different seeding distances. We added a remark regarding the usage of "real" metric units to be easily adaptable to new datasets in the conclusion.

- I could not entirely follow the rules you are applying about what temporal steps you include, and what you ignore. You seem not to be processing any image where the coverage >75%? And you also I think ignore some early data where plants are very small. Could it be that the deep learning would out-perform your approach at these times? It may not be reasonable to claim you match the deep learned method if you are ignoring the most challenging images. Or perhaps it doesnt matter if you can reliably predict plant locations in these harder images from other time steps. Please discuss this.
>>> Yes, we ignore coverages below 1% since the plant are hardly visible and above 75% because

rougly at this point, we are in the transition to canopy closure. At this growing stage, neighboring plants cannot be distinguished from each other, any more. The deep-learned approach, as the Mask R-CNN for the Cauliflower dataset, is trained on specific plant sizes, whereas our Computer Vision approach works – at least in the coverage region 1%-75% - independently of the actual plant size due to the adaptive gaussian blur and thresholding. The strength of our approach is more due to the size agnostic image extraction that can be used to train better plant recognition models in the end. We add a remark on that in conclusion section.

- It wasn't clear to me the input image to the hough transform. In the caption for Fig8, you say "applied to the rasterised image shown on the right" but I do not know what this image is.
>>> Before, the plant positions are available as point clouds. The Hough transform is a method for (rastered) image data. Thus, we have to transfer the point cloud into an image. We did this by a 2D histogram, i.e. we defined a pixel grid and count the number of points in each pixel. With this histogram image, we can finally perform the Hough transform method.

- Line 608. "Our method yields more precise plant positions than the Mask R-CNN approach". What does this mean? You are using Mask R-CNN as a ground truth, surely it is impossible to outperform it?
>>> We claimed that since with the gaussian blur, we exploit the circular shape of the plants, so that the peak detection is able to find the real center of the plant. The Mask R-CNN detects the central plant position by the center of its bounding box. If the plant has a highly non-circular shape, the bounding box center may not be on the plant stem. Additionally, the Mask R-CNN only sees one picture of the plant, whereas our approach can calculate the plant centroid by using the positions of many time steps.

- In your evaluation and discussion, you are missing an opportunity to talk in more detail about precision and recall. "For most images precision is above 90%" is extremely vague, the readers of your paper would benefit from more detail here. Recall is "of the true plants, what percentage were found?". Precision is "Of the plants found, what % were truly plants?". These are notably different, and in your recall is consistently above 90%, whereas precision is sometimes 80-90, a bit lower. This means that, informally, your algorithm is very good at finding plants, it finds some false positives. This is worth discussing in more detail.
>>> Yes, that is correct. In fact, the false positives found by our workflow are a major challenge. Mostly, they are due to double recognitions of single plants or remaining weed, since we are only able to filter what we call "off-line weed". Weed within the seeding lines is still present. We added some thoughts on that.

- What does hands-on mean in the abstract? Semi-automatic?
>>> Yes, partly. The "hands-on" should mention the semi-automatic and the modular approach. Between the steps, the user can investigate the results and may adjust the parameters to get to better results for the corresponding dataset.

Minor typos:

The spelling and grammar of the paper is fine, and understandable. There are some sentences that would benefit from a bit of proof reading. I try to list some here:

25: to extract -> the extraction of
35: delete "almost"
43: to allocate -> allocation of
46: points -> times?
76: out -> our
76: various -> a variety of
89: demands for -> requires
~215: and plants regarding a standard RGB image -> and plants compared with a standard RGB image
254: catch up -> highlight / find / obtain
Grouping the plants: chapter -> section
394: seeing -> seeding
461: procedure work -> procedure to work
>>> Thanks, we corrected the mentioned typos except the suggestion to turn „Grouping the plants" into a section. It comprises the aligning, (weed) filtering, and the clustering and, thus, is a superordinate heading.

Reviewer #2: This interesting Research Article describes a workflow for automated spatiotemporal analysis and determination of plant position of crop image data acquired using Unmanned Aerial Vehicles (UAVs). The approach is novel and of great interest from the perspective of precision agriculture. In support of the image analysis workflow, validations are provided in the form of two use case scenarios that include measuring the fungal disease Cercospora leaf spot in Sugar Beet, and also harvest prediction of Cauliflower plants. The Plant Extraction workflow is showcased in the manuscript, and an important claim made by the authors is that this method yields more precise plant positions than a Deep Learning Mask R-CNN approach.

From a reproducibility perspective, the software for the Plant Extraction workflow is publicly available in GitHub repository https://github.com/mrcgndr/plant_extraction_workflow where it has been ascribed an OSI-approved Apache-2.0 License. This GitHub repository includes a config file for the demo pipeline and an execution script. In addition, a subset of the Cercospora leaf spot dataset referred to in the manuscript has been submitted to the GigaScience DataBase and includes UAV images (TIFF format) of Sugar Beet crop data from 13 acquisition dates. The multispectral orthoimage data used for spatiotemporal analysis includes 6 channels, namely: Blue; Green; Red; RedEdge; Near-IR; and Thermal. Importantly, the Ground Truth has also been submitted to the GigaScience DataBase and includes annotated plant positions for the Sugar Beet field from 4 acquisition dates.

Major comment

A major concern is the availability of the complete imaging datasets used in the analysis. In the Data Availability section, the authors state that, "Both datasets are used for ongoing research purposes and therefore cannot be published completely". However, the authors do report that the subset of Sugar Beet image data submitted to the GigaScience DataBase are sufficient to reproduce and validate the functionality of the introduced workflow.

As the complete imaging datasets used in the analysis are Restricted Access, can the authors please outline how a researcher can gain access to these data?
>>> Thanks for that remark. Unfortunately, we are not yet able to provide the complete sugar beet dataset, as a lot of research is still being done on the IfZ side. Definitely, the complete data will be published in form of data papers in the near future. However, the cauliflower dataset has since been published in the form of a data paper. We have added the reference in the manuscript.

Reviewer #3:
1. Maybe Typo in line 76 - out should by our
>>> Thanks, corrected.

2. The explanation about the background and mentioning the problem also several approaches are well described, however, to strengthen the position of the proposed framework, kindly review the other framework that also uses the same approach or workflow
>>> We mentioned the work of Lottes et al. where they also apply some Computer Vision methods. E.g., they perform the seeding line detection with Hough transform method, as well. However, they use the complete segmentation masked image in full resolution. In our workflow, the detection plant peaks are rasterized in a lower resolution image before performing Hough transform. This makes our approach potentially faster. We added this comment in the regarding section.

3. For describing the Plant Extraction workflow (line 191), it would be better to provide a flow chart for the complete process, including several possibilities in case of the implementation's scenario in fields.
>>> Thanks for that remark. Actually, Figure 1 already represents the flowchart in form of example images connected by arrows titled with the necessary steps. We added another cross reference to Figure 1. We hope, that this resolves your concerns.

4. The proposed workflow might have prerequisites that must be met, so it would be better for providing the preprocessing step before the main flow.
>>> Thanks, good point. In our case, there is not much preprocessing done, but this, indeed, can change for custom datasets. We added a brief passage with possible preprocessing steps.

5. The application of the workflow for specific cases, for example, the Image Extraction for Disease Severity Classification, is a nice explanation for emphasizing the advantage over the existing framework. However, it would be better if it also showed the performance comparison for that task.

>>> First of all, the disease severity classification is just one conceivable use case of the dataset gained by our workflow. Thus, the focus of this work is not on the deep learning part of corresponding model. Additionally, we have neither a model nor a dataset for reference. In future research we definitely work on that topic, but for this work it is beyond the scope. The classification section should be rather considered as an inspiration for application of the introduced workflow.

6. For the conclusion, more deeply on the workflow uniqueness and mentioning the advantage, limitations, applications, and limitations for further improvement would improve the final closure.
>>> We added some passages by pointing out advantages and limitations in the conclusion.

Close
