# Supplementary material for: Agricultural plant cataloging and establishment of a data framework from UAV-based crop images by computer vision
Source: Gigascience. 2022 Jun 17;11:giac054. doi: 10.1093/gigascience/giac054 (PMC9205758; doi:10.1093/gigascience/giac054)
Supplement: giac054_Response_to_Reviewer_Comments_Revision_1 [file giac054_response_to_reviewer_comments_revision_1.pdf]

Author's Response To Reviewer Comments

Close

In the following, our response is marked with ">>>"

Reviewer reports:
Reviewer #1: The authors have addressed my concerns, and I have no further issues with this paper.

A minor grammar edit in the conclusion:

"We can directly regard to real-world measures like plant radii or seeding distance without having to consider the image resolution."

regard -> refer to

>>> Thanks, we have corrected the mistake.

Reviewer #2: I am unable to run this software tool. Of note, it requires the following dependencies:

dacite
fluidml
matplotlib
pandas
pycpd
pykml
pyproj
rasterio
scikit-image
scikit-learn
simplekml

For a software tool with this many dependencies, the authors would need to Dockerize the software application or to use a Code Ocean Capsule so that it would be easier for a reviewer to re-run their analysis.

As for the image data, I am disappointed that the authors are unwilling to provide the entire image dataset used in their analysis. The test data that has been submitted includes multispectral orthoimage data files (TIFF format), and supporting KML (Keyhole Markup Language) files with the ground truth coordinates of annotated plant positions.

I am rejecting this manuscript on the grounds of poor reproducibility. In my opinion, the authors have made little effort towards enabling a researcher to rerun their analysis in a simple manner through use of Docker or Code Ocean, or even to reuse the entire image dataset included in their analysis.

>>> We have considerably improved the reproducibility of our workflow. Although most of the dependencies are still necessary, we have made the workflow more accessible to users by following aspects:
- We noticed some complications on Windows with installing the "rasterio" python package. Therefore, we switched from pip virtual environments to conda, where no problem occurs. We have tested this installation approach on Windows and Linux
- We dockerized the workflow. However, this approach only works on Linux machines since there is no base container with conda running on Windows.
- We improved the documentation of the workflow by providing a better readme file in the GitHub repository. Both installation methods are described, so that users can run the demo pipeline out of the box. The only "external" thing needed is to download the demo dataset. We asked for the download link

from the GigaScience database an will provide it directly in the GitHub readme file.
- We added a declaration of dependencies in the readme file where we shortly note which role the respective dependency plays in our software.

We hope, the software is now executable and fulfills the necessary standards of reproducibility. The full dataset will be published in the future by data papers but due to ongoing research, we cannot publish the full dataset in the scope of this paper.

Close
