# Supplementary material for: Agricultural plant cataloging and establishment of a data framework from UAV-based crop images by computer vision
Source: Gigascience. 2022 Jun 17;11:giac054. doi: 10.1093/gigascience/giac054 (PMC9205758; doi:10.1093/gigascience/giac054)
Supplement: giac054_Response_to_Reviewer_Comments_Revision_2 [file giac054_response_to_reviewer_comments_revision_2.pdf]

Author's Response To Reviewer Comments

Close

Following concerns are no revised:

* we submitted the dataset to SciCrunch, it is not yes published there, but we have already got an RRID which we also added to the Manuscript under "Availabiliy of Source Code"
* the demo dataset is now available on GigaDB, we cited the data with its DOI
* we stated the requirements for the conda and docker installation

Close
