# Supplementary material for: Agricultural plant cataloging and establishment of a data framework from UAV-based crop images by computer vision
Source: Gigascience. 2022 Jun 17;11:giac054. doi: 10.1093/gigascience/giac054 (PMC9205758; doi:10.1093/gigascience/giac054)
Supplement: giac054_Reviewer_1_Report_Original_Submission — Michael Pound -- 12/11/2021 Reviewed [file giac054_reviewer_1_report_original_submission.pdf]

Reviewer Report

Title: Agricultural Plant Cataloging and Establishment of a Data Framework from UAV-based Crop Images by Computer Vision

Version: Original Submission **Date:** 12/11/2021

Reviewer name: Michael Pound

Reviewer Comments to Author:

The authors present an automated pipeline for identification of plants in UAV images. Recognition happens over a sequence of images, corroborating the location of plants growing over time. Overall the approach appears to perform well, and has comparable accuracy to an R-CNN based approach, which would be considered standard.

Overall I have a positive impression of the paper, but there are a few concerns I think should be addressed. The main justification of the approach in this paper is that it is effective, and demonstrably similar in performance to deep learning approaches. This is fine, it is reasonable to publish "traditional" image analysis pipelines if they work well. However there were some missing details on the robustness and specifics of the algorithm that would be very useful for readers. I list my main points below:

- As a bottom up pipeline, there are numerous thresholds and defined values that underpin the algorithm. For example, the threshold value for plant vs soil, the function to calculate the width of the gaussian blur, the valid plant region from Figure 9, and so on. While these appear effective, no detail is given on the robustness of the approach to choices of these values. It is possible (without evidence shown) that the algorithm quickly breaks with even small changes to these parameters, if this is the case then there would be a stronger argument for moving towards a deep learning approach, where robustness could be gained from training data. How did you pick these values, what was your experience of picking these values? If another reader comes along with a new dataset, how easy will it be for them to apply your pipeline?
- Related to the above, you do not mention if there are any changes to the experimental parameters moving from one dataset to the cauliflower dataset. If your approach worked immediately with no changes, that would obviously be a postitive!
- I could not entirely follow the rules you are applying about what temporal steps you include, and what you ignore. You seem not to be processing any image where the coverage >75%? And you also I think ignore some early data where plants are very small. Could it be that the deep learning would out-perform your approach at these times? It may not be reasonable to claim you match the deep learned method if you are ignoring the most challenging images. Or perhaps it doesnt matter if you can reliably predict plant locations in these harder images from other time steps. Please discuss this.
- It wasn't clear to me the input image to the hough transform. In the caption for Fig8, you say "applied to the rasterised image shown on the right" but I do not know what this image is.
- Line 608. "Our method yields more precise plant positions than the Mask R-CNN approach". What does this mean? You are using Mask R-CNN as a ground truth, surely it is impossible to outperform it?
- In your evaluation and discussion, you are missing an opportunity to talk in more detail about precision

and recall. "For most images precision is above 90%" is extremely vague, the readers of your paper would benefit from more detail here. Recall is "of the true plants, what percentage were found?". Precision is "Of the plants found, what % were truly plants?". These are notably different, and in your recall is consistently above 90%, whereas precision is sometimes 80-90, a bit lower. This means that, informally, your algorithm is very good at finding plants, it finds some false positives. This is worth discussing in more detail.

- What does hands-on mean in the abstract? semi-automatic?

Minor typos:

The spelling and grammar of the paper is fine, and understandable. There are some sentences that would benefit from a bit of proof reading. I try to list some here:

25: to extract -> the extraction of
35: delete "almost"
43: to allocate -> allocation of
46: points -> times?
76: out -> our
76: various -> a variety of
89: demands for -> requires
~215: and plants regarding a standard RGB image -> and plants compared with a standard RGB image
254: catch up -> highlight / find / obtain
Grouping the plants: chapter -> section
394: seeing -> seeding
461: procedure work -> procedure to work

Methods

Are the methods appropriate to the aims of the study, are they well described, and are necessary controls included? Choose an item.

Conclusions

Are the conclusions adequately supported by the data shown? Choose an item.

Reporting Standards

Does the manuscript adhere to the journal's guidelines on minimum standards of reporting? Choose an item.

Choose an item.

Statistics

Are you able to assess all statistics in the manuscript, including the appropriateness of statistical tests used? Choose an item.

Quality of Written English

Please indicate the quality of language in the manuscript: Choose an item.

Declaration of Competing Interests

Please complete a declaration of competing interests, considering the following questions:

- Have you in the past five years received reimbursements, fees, funding, or salary from an organisation that may in any way gain or lose financially from the publication of this manuscript, either now or in the future?
- Do you hold any stocks or shares in an organisation that may in any way gain or lose financially from the publication of this manuscript, either now or in the future?
- Do you hold or are you currently applying for any patents relating to the content of the manuscript?
- Have you received reimbursements, fees, funding, or salary from an organization that holds or has applied for patents relating to the content of the manuscript?
- Do you have any other financial competing interests?
- Do you have any non-financial competing interests in relation to this paper?

If you can answer no to all of the above, write 'I declare that I have no competing interests' below. If your reply is yes to any, please give details below.

I declare that I have no competing interests

I agree to the open peer review policy of the journal. I understand that my name will be included on my report to the authors and, if the manuscript is accepted for publication, my named report including any attachments I upload will be posted on the website along with the authors' responses. I agree for my report to be made available under an Open Access Creative Commons CC-BY license (http://creativecommons.org/licenses/by/4.0/). I understand that any comments which I do not wish to be included in my named report can be included as confidential comments to the editors, which will not be published.

Choose an item.

To further support our reviewers, we have joined with Publons, where you can gain additional credit to further highlight your hard work (see: https://publons.com/journal/530/gigascience). On publication of this paper, your review will be automatically added to Publons, you can then choose whether or not to claim your Publons credit. I understand this statement.

Yes Choose an item.
