# Supplementary material for: Agricultural plant cataloging and establishment of a data framework from UAV-based crop images by computer vision
Source: Gigascience. 2022 Jun 17;11:giac054. doi: 10.1093/gigascience/giac054 (PMC9205758; doi:10.1093/gigascience/giac054)
Supplement: giac054_Reviewer_2_Report_Original_Submission — Chris Armit -- 12/13/2021 Reviewed [file giac054_reviewer_2_report_original_submission.pdf]

Reviewer Report

Title: Agricultural Plant Cataloging and Establishment of a Data Framework from UAV-based Crop Images by Computer Vision

Version: Original Submission **Date:** 12/13/2021

Reviewer name: Chris Armit

Reviewer Comments to Author:

This interesting Research Article describes a workflow for automated spatiotemporal analysis and determination of plant position of crop image data acquired using Unmanned Aerial Vehicles (UAVs). The approach is novel and of great interest from the perspective of precision agriculture. In support of the image analysis workflow, validations are provided in the form of two use case scenarios that include measuring the fungal disease Cercospora leaf spot in Sugar Beet, and also harvest prediction of Cauliflower plants. The Plant Extraction workflow is showcased in the manuscript, and an important claim made by the authors is that this method yields more precise plant positions than a Deep Learning Mask R-CNN approach.

From a reproducibility perspective, the software for the Plant Extraction workflow is publicly available in GitHub repository https://github.com/mrcgndr/plant_extraction_workflow where it has been ascribed an OSI-approved Apache-2.0 License. This GitHub repository includes a config file for the demo pipeline and an execution script. In addition, a subset of the Cercospora leaf spot dataset referred to in the manuscript has been submitted to the GigaScience DataBase and includes UAV images (TIFF format) of Sugar Beet crop data from 13 acquisition dates. The multispectral orthoimage data used for spatiotemporal analysis includes 6 channels, namely: Blue; Green; Red; RedEdge; Near-IR; and Thermal. Importantly, the Ground Truth has also been submitted to the GigaScience DataBase and includes annotated plant positions for the Sugar Beet field from 4 acquisition dates.

Major comment

A major concern is the availability of the complete imaging datasets used in the analysis. In the Data Availability section, the authors state that, "Both datasets are used for ongoing research purposes and therefore cannot be published completely". However, the authors do report that the subset of Sugar Beet image data submitted to the GigaScience DataBase are sufficient to reproduce and validate the functionality of the introduced workflow.

As the complete imaging datasets used in the analysis are Restricted Access, can the authors please outline how a researcher can gain access to these data?

Methods

Are the methods appropriate to the aims of the study, are they well described, and are necessary controls included? Choose an item.

Conclusions

Are the conclusions adequately supported by the data shown? Choose an item.

Reporting Standards

Does the manuscript adhere to the journal's guidelines on minimum standards of reporting? Choose an item.

Choose an item.

Statistics

Are you able to assess all statistics in the manuscript, including the appropriateness of statistical tests used? Choose an item.

Quality of Written English

Please indicate the quality of language in the manuscript: Choose an item.

Declaration of Competing Interests

Please complete a declaration of competing interests, considering the following questions:

- Have you in the past five years received reimbursements, fees, funding, or salary from an organisation that may in any way gain or lose financially from the publication of this manuscript, either now or in the future?
- Do you hold any stocks or shares in an organisation that may in any way gain or lose financially from the publication of this manuscript, either now or in the future?
- Do you hold or are you currently applying for any patents relating to the content of the manuscript?
- Have you received reimbursements, fees, funding, or salary from an organization that holds or has applied for patents relating to the content of the manuscript?
- Do you have any other financial competing interests?
- Do you have any non-financial competing interests in relation to this paper?

If you can answer no to all of the above, write 'I declare that I have no competing interests' below. If your reply is yes to any, please give details below.

I work for GigaScience where I perform checks on the quality of image data and the image analysis approach. My funding is not dependent on the outcome of this review, and I declare that I have no competing interests.

I agree to the open peer review policy of the journal. I understand that my name will be included on my report to the authors and, if the manuscript is accepted for publication, my named report including any attachments I upload will be posted on the website along with the authors' responses. I agree for my

report to be made available under an Open Access Creative Commons CC-BY license (http://creativecommons.org/licenses/by/4.0/). I understand that any comments which I do not wish to be included in my named report can be included as confidential comments to the editors, which will not be published.

Choose an item.

To further support our reviewers, we have joined with Publons, where you can gain additional credit to further highlight your hard work (see: https://publons.com/journal/530/gigascience). On publication of this paper, your review will be automatically added to Publons, you can then choose whether or not to claim your Publons credit. I understand this statement.

Yes Choose an item.
