# Supplementary material for: Agricultural plant cataloging and establishment of a data framework from UAV-based crop images by computer vision
Source: Gigascience. 2022 Jun 17;11:giac054. doi: 10.1093/gigascience/giac054 (PMC9205758; doi:10.1093/gigascience/giac054)
Supplement: giac054_Reviewer_2_Report_Revision_2 — Chris Armit -- 5/10/2022 Reviewed [file giac054_reviewer_2_report_revision_2.pdf]

Reviewer Report

Title: Agricultural Plant Cataloging and Establishment of a Data Framework from UAV-based Crop Images by Computer Vision

Version: Revision 2 **Date:** 5/10/2022

Reviewer name: Chris Armit

Reviewer Comments to Author:

This Research Article describes a workflow for automated spatiotemporal analysis and determination of plant position of crop image data acquired using Unmanned Aerial Vehicles (UAVs). The Plant Extraction workflow is showcased in the manuscript, and an important claim made by the authors is that this method yields more precise plant positions than a Deep Learning Mask R-CNN approach.
The software for the Plant Extraction workflow is publicly available in GitHub repository https://github.com/mrcgndr/plant_extraction_workflow where it has been ascribed an OSI-approved Apache-2.0 License. In addition, a subset of the Cercospora leaf spot dataset referred to in the manuscript has been submitted to the GigaScience DataBase and includes multispectral image and annotated plant positions from 4 acquisition dates.
When I previously reviewed this manuscript, I was concerned about how easy it was for a researcher to rerun the analysis, and towards this end I questioned whether the workflow could be containerised. I am delighted to report that the authors have now containerised the workflow. Of note, the authors highlight that the Dockerised workflow package only works on LINUX. As I do not have a LINUX machine, I was unable to test how easy it is to run the workflow package. However, Reviewer 1 has confirmed that the Dockerised workflow package runs on LINUX, and based on this confirmation I recommend this manuscript for publication in GigaScience.

Methods

Are the methods appropriate to the aims of the study, are they well described, and are necessary controls included? Choose an item.

Conclusions

Are the conclusions adequately supported by the data shown? Choose an item.

Reporting Standards

Does the manuscript adhere to the journal's guidelines on [minimum standards of reporting?]() Choose an item.

Choose an item.

Statistics

Are you able to assess all statistics in the manuscript, including the appropriateness of statistical tests used? Choose an item.

Quality of Written English

Please indicate the quality of language in the manuscript: Choose an item.

Declaration of Competing Interests

Please complete a declaration of competing interests, considering the following questions:

- Have you in the past five years received reimbursements, fees, funding, or salary from an organisation that may in any way gain or lose financially from the publication of this manuscript, either now or in the future?
- Do you hold any stocks or shares in an organisation that may in any way gain or lose financially from the publication of this manuscript, either now or in the future?
- Do you hold or are you currently applying for any patents relating to the content of the manuscript?
- Have you received reimbursements, fees, funding, or salary from an organization that holds or has applied for patents relating to the content of the manuscript?
- Do you have any other financial competing interests?
- Do you have any non-financial competing interests in relation to this paper?

If you can answer no to all of the above, write 'I declare that I have no competing interests' below. If your reply is yes to any, please give details below.

I work for GigaScience where I perform checks on the quality of image data and the image analysis approach. My funding is not dependent on the outcome of this review, and I declare that I have no competing interests.

I agree to the open peer review policy of the journal. I understand that my name will be included on my report to the authors and, if the manuscript is accepted for publication, my named report including any attachments I upload will be posted on the website along with the authors' responses. I agree for my report to be made available under an Open Access Creative Commons CC-BY license (http://creativecommons.org/licenses/by/4.0/). I understand that any comments which I do not wish to be included in my named report can be included as confidential comments to the editors, which will not be published.

Choose an item.

To further support our reviewers, we have joined with Publons, where you can gain additional credit to further highlight your hard work (see: https://publons.com/journal/530/gigascience). On publication of this paper, your review will be automatically added to Publons, you can then choose whether or not to claim your Publons credit. I understand this statement.

Yes Choose an item.
